# Synthetic two-species allodiploid and three-species allotetraploid *Saccharomyces* hybrids with euploid (complete) parental subgenomes

**DOI:** 10.1038/s41598-023-27693-2

**Published:** 2023-01-20

**Authors:** Zsuzsa Antunovics, Adrienn Szabo, Lina Heistinger, Diethard Mattanovich, Matthias Sipiczki

**Affiliations:** 1 Department of Genetics and Applied Microbiology, University of Debrecen, Debrecen, Hungary; 2 Department of Biotechnology, Institute of Microbiology and Microbial Biotechnology, University of Natural Resources and Life Sciences Vienna (BOKU), Muthgasse 18, 1190 Vienna, Austria

**Keywords:** Genetics, Microbiology

## Abstract

Combination of the genomes of *Saccharomyces* species has great potential for the construction of new industrial strains as well as for the study of the process of speciation. However, these species are reproductively isolated by a double sterility barrier. The first barrier is mainly due to the failure of the chromosomes to pair in allodiploid meiosis. The second barrier ensures that the hybrid remains sterile even after genome duplication, an event that can restore fertility in plant interspecies hybrids. The latter is attributable to the autodiploidisation of the allotetraploid meiosis that results in sterile allodiploid spores (return to the first barrier). Occasionally, mating-competent alloaneuploid spores arise by malsegregation of *MAT*-carrying chromosomes. These can mate with cells of a third species resulting in aneuploid zygotes having at least one incomplete subgenome. Here we report on the construction of euploid three-species hybrids by making use of “rare mating” between a sterile *S. kudriavzevii* x *S. uvarum* allodiploid hybrid and a diploid *S. cerevisiae* strain. The hybrids have allotetraploid 2n^Sc^n^Sk^ n^Su^ genomes consisting of complete sets of parental chromosomes. This is the first report on the production of euploid three-species *Saccharomyces* hybrids by natural mating, without genetic manipulation. The hybrids provide possibilities for studying the interactions of three allospecific genomes and their orthologous genes present in the same cell.

## Introduction

The genus *Saccharomyces* comprises eight “natural species”, namely *S. arboricola, S. cerevisiae, S. eubayanus, S. jurei, S. kudriavzevii, S. mikatae, S. paradoxus,* and *S. uvarum* (recently reviewed by Alsammar and Delneri^1^) and many strains of chimeric (admixed) genomes that are, somewhat superficially, also called “interspecies hybrids”. Two groups of the chimeric, mostly brewing strains of highly diverse genome structures are accommodated in the so-called “hybrid species” *S. bayanus* and *S. pastorianus* (*S. carlsbergensis*) (e.g.^1–5^). The chimeric strains identified in other environments (e.g. in wine-making processes) are not grouped in separate species and are assumed to have evolved from hybrids of natural species by loss and rearrangements of mosaics in the parental subgenomes (for a review, see e.g.^6^).

The taxonomic division of the genus was mainly based on the biological species concept and later confirmed by the analysis of barcode and genome sequences. In the biological species concept introduced to *Saccharomyces* taxonomy by Naumov^7^, the species are populations of interbreeding strains isolated by sterility barriers. While conspecific strains form fertile hybrids (producing functional gametes), the strains that belong to different species either do not form hybrids (prezygotic sterility barrier) or their hybrids do not form functional gametes (ascospores) (postzygotic sterility barrier). The *Saccharomyces* species are isolated by postzygotic sterility barriers. All *Saccharomyces* species can form viable (allodiploid) hybrids with any other *Saccharomyces* species but the hybrids either do not sporulate or their spores are not viable.

Allodiploid sterility is mainly due to the failure of the chromosomes of the subgenomes to pair in meiosis I (e.g.^8–11^) which results in the abruption of the meiotic process (“first sterility barrier”). In plants, allodiploid sterility can be circumvented by genome duplication, which “diploidises” the subgenomes (for a review, see^12^). In the autodiploid subgenomes of the allotetraploid plant hybrid, each chromosome has a homologous partner to pair with, which allows successful meiosis. The allodiploid plant gametes produced in the allotetraploid meiosis are functional and can mate with other gametes to form allotri-, allotetra- and even allopolyploid zygotes (hybrids) depending on the ploidy of the partner gamete^13^.

Genome duplication also occurs in yeast interspecies hybrids^14–17^ and the resulting allotetraploid hybrids also produce viable allodiploid gametes (ascospores capable of germination). Their viability is frequently misinterpreted as the breach of the sterility barrier by genome duplication^18^. However, in contrast to the allodiploid gametes of the plant hybrids, the allodiploid ascospores cannot function as gametes^14,19^. This difference between the plant hybrids and the *Saccharomyces* hybrids is attributable to the different mechanisms of the regulation of sexual processes in plants and yeasts.

In *Saccharomyces*, the *MAT* cassettes in the *MAT* loci determine which of the alternative sexual programmes, mating-fertilisation or meiosis-sporulation, is active. In a haploid genome there is only one *MAT* locus, which contains either a *MATa* or a *MATalpha* cassette. Single copies of *MAT* cassettes allow mating but repress meiosis-sporulation. Two haploid cells having different cassettes in their *MAT* loci can mate (conjugate) and form a heterozygous *MATa/MATalpha* diploid. *MATa/MATalpha* heterozygosity blocks the mating programme and mating-type switching but allows meiosis^20^. However, in spite of the activation of the meiotic programme, the allodiploid cells are prevented from producing viable and functional haploid ascospores (gametes) by the first sterility barrier. If the allodiploid genome of the hybrid becomes allotetraploid by spontaneous genome duplication, it will be able to produce viable allodiploid ascopores like the allotetraploid plants, however, in contrast to the plant allodiploid gametes, the yeast allodiploid ascospores are sterile. They cannot mate because of their *MATa/MATalpha* heterozygosity (“second sterility barrier”)^19^. The two sterility barriers (the double sterility barrier) ensure the reproductive (biological) isolation of the *Saccharomyces* species. Due to the second sterility barrier, which has no counterpart in plants, the interspecies *Saccharomyces* hybrids remain sterile even upon whole-genome duplication.

Because of their sterility, the alloploid hybrids can mate neither with the parental strains nor with strains of other species. Thus, allodiploid sterility prevents both introgressive backcrosses with parental strains and hybridisation with a third species. To overcome this obstacle, two “natural”, non-GMO strategies have been proposed for hybridising of a two-species hybrid with a third species^21^.

One strategy is based on the occasional breakdown of the second sterility barrier by the loss of *MAT* heterozygosity due to occasional inaccurate distribution of chromosomes during allotetraploid meiosis. If a spore receives a *MAT*-carrying chromosome only from one of the subgenomes (los of *MAT*-heterozygosity), it becomes mating-competent. In this alloaneuploid spore (nullisomic for the *MAT*-carrying chromosome in one of the subgenomes) the mating programme is released from repression^14^. This spore can mate with a haploid cell of opposite mating activity, but the hybrid is not triploid but only aneuploid (segmental triploid), nullisomic for the chromosome lost during meiosis. Since additional parental chromosomes can also be lost during meiosis, these hybrids only have mosaic genomes consisting of aneuploid subgenomes^15^. If the mating partner is a cell of a third species, the hybrid will be a segmental allotriploid (alloaneuploid). The second sterility barrier can also be overcome by integrating genetically modified drug-inducible *HO* genes (*HO* codes for a mating-type switching endonuclease) in the genomes of the parental strains before hybridisation. The “artificial” (drug-induced) expression of these genes reactivates the mating-type switching process normally repressed by the *MATa/MATalpha* heterozygosity and makes certain hybrid cells homozygous and thus mating-competent. However, the hybrids obtained in this way also had chimeric genomes consisting of mosaics of the parental genomes^22^. The failure to produce polyploids with alloeuploid genomes can be attributed to the instability of the hybrid genomes that can easily loose chromosomes during mitotic and meiotic divisions of the hybrid cells by the “postzygotic” processes designated GARMi (Genome Autoreduction in Mitosis) and GARMe (Genome Autoreduction in Meiosis), respectively^6^. Both types of events reduce the size of one or the other subgenome. Thus, “true” allopolyploid hybrids possessing entire parental genomes cannot be produced with genetic manipulation of *HO* expression either. It has to be mentioned that the *MAT* heterozygosity of the sterile alloploid hybrids can also be broken down during the vegetative (mitotic) propagation of the hybrid cells, albeit at much lower rates than during allopolyploid meiosis. Mating-competent segregants can be formed by occasional unequal distribution of the *MAT*-carrying chromosomes between the sister nuclei (deletion of a chromosome, occurring in GARMi, see below) and by the rarely occurring mitotic gene conversion between the *MAT* loci of the subgenomes (loss of *MAT* heterozygosity)^6^.

The other natural strategy proposed in reference^21^ is based on the rare mating of sterile diploid cells. Gunge et al.^23^ described the phenomenon referred to as “rare mating” in *S. cerevisiae*. They noticed that in *MATa/MATalpha S. cerevisiae* diploid cultures, very rarely, certain cells escaped the block of the mating programme and conjugated with mating-competent cells or spores of other strains. In a previous study, we managed to make use of this phenomenon to hybridise sterile two-species kudvarum (*S. kudriavzevii* x *S. uvarum*) hybrids with *S. cerevisiae* strains to obtain three-species cekudvarum (*S. cerevisiae* x *S. kudriavzevii* x *S. uvarum*) hybrids without gene manipulation^19^. We found that the hybrids had repressed mating and mating-type switching programmes but we did not examine their genome structures. In the current work, we present data demonstrating that the three-species hybrids created in this natural way have euploid subgenomes consisting of complete parental sets of chromosomes. Since the hybrids do not form viable ascospores, their genomes are stable. This is the first report on constructing stable euploid three-species *Saccharomyces* hybrids. Interestingly, the mitochondrial genomes were uniparentally inherited.

## Methods

### Strains and culture media

All strains used in this study are listed in Table 1. The medium used for the maintenance of the parental strains 10–170, 10–1651 and 10–1653 was YEA (yeast extract glucose agar). Hybrids were isolated and maintained on MMA (minimal medium agar) or on MMA supplemented with uracil. Mating tests were performed on YEA plates. Sporulation was tested on acetate SPA (sporulation agar) plates. Cultures for DNA isolation, karyotyping and flocculation tests were grown in YEL (YEA without agar). The composition of these media was described previously^24,25^. For FACS analysis, cells were propagated in YPD (YEL supplemented with 2% peptone).

**Table 1:** List of strains.

Identification number	Strain	Genotype/phenotype	Source
10–170	*Saccharomyces cerevisiae* X4005-11A	*MATa*^*Sc*^* ho*^*Sc*^* leu2*^*Sc*^	^24^
10–1651	*Saccharomyces uvarum* JRY9193 SSS111	*MATalpha*^*Su*^* ade2*^*Su*^* ura3*^*Su*^* ho*^*Su*^	^29^
10–1653	*Saccharomyces kudriavzevii* FM1193 SSS411	*MATa*^*Sk*^* trp1*^*Sk*^* ura3*^*Sk*^* ho*^*Sk*^	^29^
II/6	Two-species kudvarum hybrids produced by mass-mating of 10–1651 and 10–1653	*MATa*^*Sk*^*/MATalpha*^*Su*^* ho*^*Sk*^*/ho*^*Su*^* ADE2*^*Sk*^*/ade2*^*Su*^* trp1*^*Sk*^*/TRP1*^*Su*^* ura3*^*Sk*^*/ura3*^*Su*^	^19^
II/6.1, II/6.2, II/6.3, II/6.4, II/6.5	Three-species cekudvarum hybrids produced by massmating of 10–170 and II/6	*MATa*^*Sc*^*/ MATa*^*Sk*^*/MATalpha*^*Su*^* ho*^*Sc*^*/ho*^*Sk*^*/ho*^*Su*^* ADE2*^*Sc*^*/ADE2*^*Sk*^*/ade2*^*Su*^* TRP1*^*Sc*^*/trp1*^*Sk*^*/TRP1*^*Su*^* URA3*^*Sc*^*/ura3*^*Sk*^*/ura3*^*Su*^* leu2*^*Sc*^*/LEU2*^*Sk*^*/LEU2*^*Su*^	^19^ and this study

### Hybridisation

Hybridisation was based on complementation of auxotrophic markers. Two-species kudvarum hybrids were obtained by mating 10–1653 *S. kudriavzevii* with 10–1651 *S. uvarum* and selection of colonies growing on MMA supplemented with uracil. Since both parental strains were ura3^−^, the two-species hybrids were auxotrophic for uracil. Their uracil auxotrophy was exploited at the construction of three-species cekudvarum hybrids. One of the kudvarum hybrids (II.6) was mated with 10–170 *S. cerevisiae leu2* and prototrophic colonies were selected on MMA plates. Hybridisation was performed in two ways: by mass-mating of cells of exponential-phase cultures^19^ and by a two-step replica-plating method^26^. Individual colonies (as products of individual zygotes) were isolated from the plates and stored at − 80 °C to prevent postzygotic changes in the genomes and segregation.

### DNA content measurement

For DNA staining, cells were grown in YPD medium at 25 °C overnight and fixed with 70% ethanol. After a washing step with PBST (PBS with Tween 20, 1:1000) the cells were incubated with RNase A (1 mg/ml) for 1 h, followed by incubation with lyticase (0.125 U/µL) for 18 min. Before staining, the cells were washed with PBST, resuspended in PBS and sonicated to avoid clumping. Propidium iodide was added at a final concentration of 100 μM and forward scatter, side scatter and propidium iodide fluorescence (488 nm/ 690 nm BP50) of 10,000 events per sample were immediately recorded on a CytoFLEX flow cytometer (Beckman Coulter). Data analysis was performed using the CytExpert analysis software (Beckman Coulter).

### Electrophoretic karyotyping and Southern analysis

Chromosomal DNA was prepared in agarose plugs as described previously^27^. Plugs were washed in TE and inserted into wells of 1.1% agarose (Chromosomal grade, Bio-Rad) gel prepared in 0.5 × TBE buffer. The chromosomes were separated by pulse-field electrophoresis in 0.5 × TBE with a CHEF-Mapper apparatus (Bio-Rad). The running parameters were: 200 V, linear ramping from 40 to 120 s for 26 h at 14 °C. The chromosomal bands were visualized by staining with ethidium-bromide and destaining in sterile water. DNA blotting on positively charged nylon transfer membrane (GE Healthcare) was performed as described before^24^. Y’ sequence PCR product was labelled with DIG High Prime DNA Labeling and Detection Starter KitII (Roche). The labelled DNA was hybridised to the membrane overnight at 68 °C after 30 min prehybridisation. After hybridisation the membrane was washed first at room temperature in 2 × SSC, 0.1% SDS and then twice at 68 °C in 0.1 × SSC, 0.1% SDS.

### PCR RFLP of marker genes

**Figure 1:**
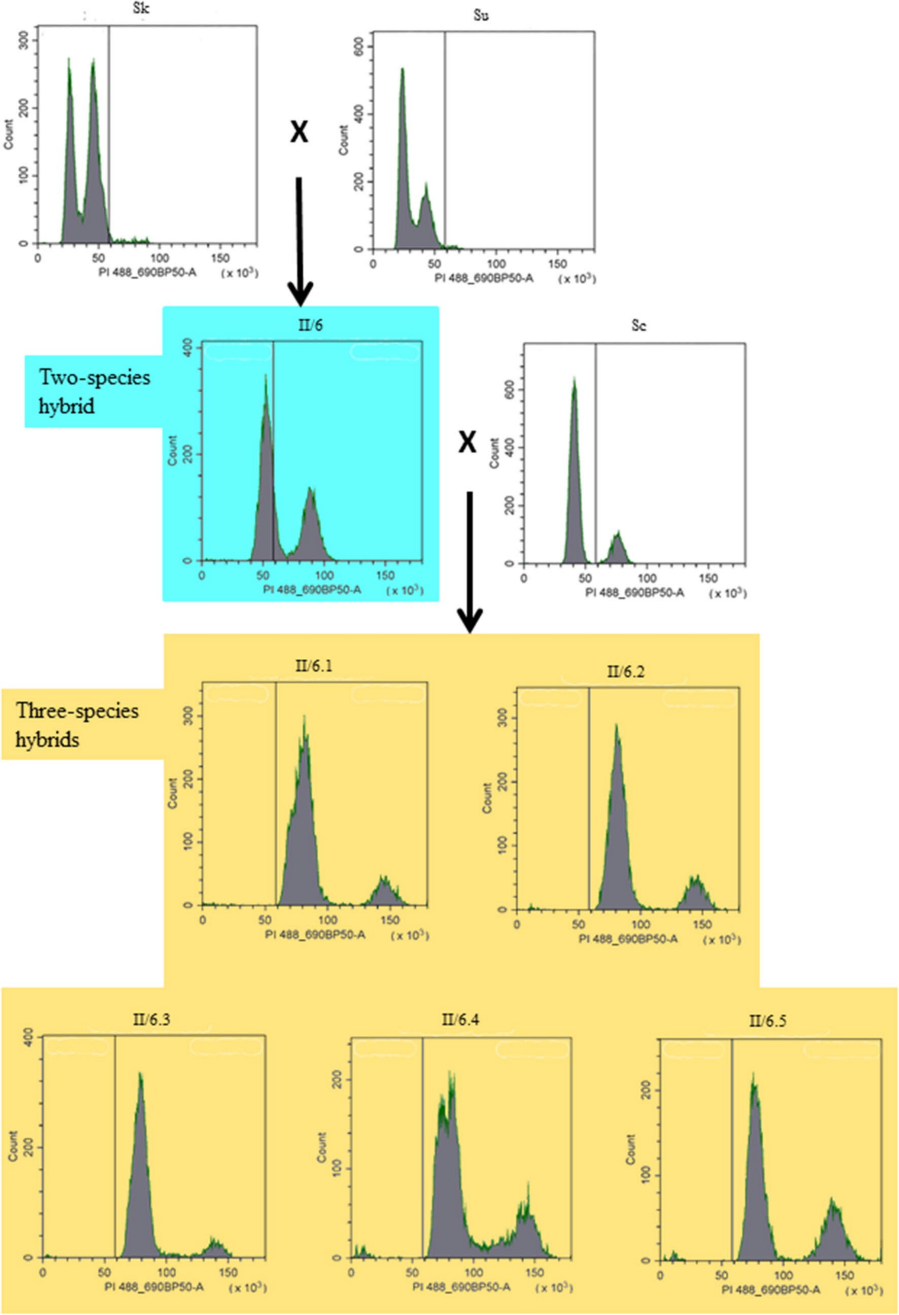
DNA content of strains measured by propidium-iodide staining and flow cytometry analysis.

The presence of the chromosomes of the parental strains was confirmed with PCR–RFLP of selected “marker” genes (Table 2; Figs. 1 and 1S). The gene-specific amplification primers are listed in Table 3. For the amplification of the marker sequences, genomic DNA was isolated from 50-ml overnight cultures grown in YEL at 26 °C^24^. For the differentiation of the genes of the parental genomes, the amplified fragments were digested with restriction endonucleases that generated specific restriction patterns for each orthologue of each gene (Table 1S). The number and size of the subfragments generated by the digestion were determined by electrophoresis in 1.4% agarose gel, 0.5 × TBE.

**Table 2:** List of markers used for the identification of parental chromosomes in the hybrid genomes.

Marker (gene)			Location in genome^1^					
			*S. cerevisiae* S288c		*S. kudriavzevii* IFO 1802		*S. uvarum* CBS7001^2^	
			Chr	Position	Chr	Position	Chr	Position
*BUD14*		YAR014C	I	166,742–168,871	1	1.75	1	1.82
*SWH1*		YAR042W	I	192,619–196,185	1	1.91	1	1.110
*CDC27*		YBL084C	II	2.57	2	2.24_	2	2.34
*CHS2*		YBR038W	II	2.206	2	2.150_	**4**	**4.270_**
*OPY1*		YBR129C	II	2.312	2	2.257	**4**	**4.377**
*FUS1*		YCL027W	III	3.50	3	3.35	3	3.186
*HCM1*		YCR065W	III	3.176	3	3.139	3	3.103
*KIN82*		YCR091W	III	3.200	3	3.165	3	3.128
*MAT*		YSC0046	III	3.156	3	3.119	3	3.79
*UGA3*		YDL170W	IV	4.88	4	4.87	4	4.76
*SNF1*		YDR477W	IV	4.843	4	4.751	**2**	**2.651**
*AFG1*		YEL052W	V	5.31	5	5.37	5	5.17
*BCK2*		YER167W	V	5.333	5	5.306	5	5.301
*STE2*		YFL026W	VI	6.57	6	6.44	6	6.32
*GSY1*		YFR015C	VI	6.120	6	6.88	6	6.77
*MNT2*		YGL257C	VII	12,481–14,157	7	7.10	**5**	**5.322**
*GND2*		YGR256W	VII	7.629	7	7.590	7	7.554
*OCA5*		YHL029C	VIII	8.33	8	8.15	8	8.22
*GND1*		YHR183W	VIII	8.299	8	8.246	**15**	**15.384**
*UBP7*		YIL156W	IX	47,292–47,693	9	9.12	9	9.30
*DAL4*		YIR028W	IX	408,468–410,375	9	9.209	9	9.244
*ECM25*		YJL201W	X	10.32	10	10.14	**6**	**6.281**
*CYR1*		YJL005W	X	10.258	10	10.218	**12**	**12.76**
*PTR2*		YKR093W	XI	11.359	11	11.331	11	11.329
*STE6*		YKL209C	XI	11.18	11	11.13	**11**	**11.13**
*MAG2*		YLR427W	XII	12.627	12	12.520	**10**	**10.544**
*LEU3*		YLR451W	XII	12.651	12	12.544	**10**	**10.569**
*CAT8*		YMR280C	XIII	13.518	13	13.452	13	13.468
*TDA1*		YMR291W	XIII	13.534	13	13.465	13	13.481
*LEM3*		YNL323W	XIV	14.22	14	14.17	14	14.17
*MET2*		YNL277W	XIV	14.78	14	14.64	14	14.66
*LRO1*		YNR008W	XIV	14.385	14	14.339	14	14.361
*ATF1*		YOR377W	XV	15.625	15	15.544	**8**	**8.4**
*RDR1*		YOR380W	XV	15.627	15	15.546	**8**	**8.436**
*SAM3*		YPL274W	XVI	16.18	16	16.3	16	16.31
*PRP4*		YPR178W	XVI	16.535	16	16.482	16	16.510

^1^In reference^29^ or in the *Saccharomyces* Genome Database (SGD): https://www.yeastgenome.org/.

^2^Genes located on chromosomes that are not counterparts of the *S. cerevisiae* chromosomes are shown on bold font.

**Table 3:** List of primers used for the amplification of chromosomal markers.

Marker/gene	Species^1^	Primer		
		Name	Sequence	Source
*AFG1*	*S.c.*, *S.u*	AFG1F	TTTCAAGTCACTGACGTGGCA	^35^
		AFG1R	CATCTGCGATTTCTTGGCAA	
	*S.k*	AFG1kF	TGTCGGTATCAATCTACGAACGT	This study
		AFG1kR	GGAAGTAGTAGACTGACGATACTGGT	
*ATF1*	*S.c.*, *S.u.*, *S.k*	ATF1F	TGGAAAAAATTTATATTTGTATCTAATCATTGTATG	^36^
		ATF1R	CCAATGAAAATGCCTGATGCCA	
*BCK2*	*S.c.*, *S.u*	BCK2F	TAGAAAACGAGCCAACACTGG	^35^
		BCK2R	CTCAATCCCAATCCCGTATT	
	*S.k*	BCK2kR	ATTGAGATCTGATAAAATGA	This study
*BUD14*	*S.c.*, *S.u.*, *S.k*	BUD14F	TGAATTGTTGGAAAAATGAAAACATG	^36^
		BUD14R	CGAATAATTTCATCCAATTGCTTCAT	
*CAT8*	*S.c.*, *S.u.*, *S.k*	CAT8F	TCCAATATTAGTATCAACAACTTTCTATAYCARAAYGA	^36^
		CAT8R	CTACTTGGCRTTTTGCCAYTGRAA	
*CDC27*	*S.c.*, *S.u*	CDC27F	GCATCTTTTTTCCTCCCAACT	^35^
		CDC27R	ACGCTGCCTGAAATCATGTAT	
	*S.k*	CDC27kF	TGTAACTCATCAATAACAAC	This study
*CHS2*	*S.c., S.u., S.k*	CHS2F	AACCATCCAACAAGACAGCA	^35^
		CHS2R	GCGACCAATTCCCAACAAA	
*CYR1*	*S.c.*, *S.u.*, *S.k*	CYR1F	CTACGAAGGAAAGTGTCCTCTTTRGTTCGTGG	^36^
		CYR1R	CCGTGTGTAGAATTTAGTGTAGAATTGACRGC	
*DAL4*	*S.c.*, *S.u*	DAL4F	CAGAGACTTGAAACCGGTTGA	^35^
		DAL4R	ATACATAGAGCCATTGCCACA	
	*S.k*	DAL4kR	CATATCAGCACCACATGCTAACG	This study
*ECM25*	*S.c.*, *S.u*	ECM25F	ATGAAATTGCCACAGGCAC	^35^
		ECM25R	TCATCAACAATTGGTAACGGA	
	*S.k*	ECM25kR	GTTTCTCCTTACATAGAGCTGG	This study
*FUS1*	*S.c.*, *S.u*	FUS1F	ACCGCAGCATATACTGACACC	^35^
		FUS1R	ACTTTTTCACCCAGCGAGAT	
	*S.k*	FUS1kF	CGACAACAACTGTGATGACGAC	This study
		FUS1kR	TGAAATATGTAGAACCTCTCAAGAACC	
*GND1*	*S.c.*, *S.u*	GND1F	ATCTTTGATGCCAGGTGGTT	^35^
		GND1R	TTGGCTGGCAATCTTTCAGA	
	*S.k*	GND1F	GGTGATATGCAGTTGATTTGC	This study
		GND1kR	TGATGTGAATATCTTTGTC	
*GND2*	*S.c.*, *S.u*	GND2F	GGTGATATGCAGTTGATTTGC	^35^
		GND2R	GATATATTACCTCCGTGCCCA	
	*S.k*	GND2kR	TGATGTGAATATCTTTGTC	This study
*GSY1*	*S.c.*, *S.u.*, *S.k*	GSY1F	ATTGGAAAAAGAATTTTCGAGCACACRATGAG	^36^
		GSY1R	AATTTCTTGCCACCGGCAAGGGTATTCATATT	
*HCM1*	*S.c.*, *S.u.*, *S.k*	HCM1F	CCAAGAGAACACTAGAAGACGAAAAGGAAA	This study
		HCM1R	GTCGTGATATACCTGATTGGAGTCTCTAT	
*KIN82*	*S.c.*, *S.k*	KIN82F	GCCCTGAAAGTTTTGAGTAAACAYGARATGAT	^36^
		KIN82R	TCGTCATCATTTGCAACTTTCTCRCARAACAT	
	*S.u*	KIN82uF	GCCTTGAAAGTATTAAGTAAGCATGAAATGA	This study
*LEM3*	*S.c.*, *S.u*	LEM3F	AGCCTGTGCGTACAAAGAACA	^35^
		LEM3R	AATGGATTTCTACCGCCAA	
	*S.k*	LEM3kF	CAGAAGAAGAAGAAGATGTCGA	This study
		LEM3kR	TGATAAAGTCGATGCATCAG	
*LEU3*	*S.c.*, *S.u*	LEURF	TTAAGCGCCGACACTTCGT	^35^
		LEU3R	CCATATGCTTCGCATTATTCC	
	*S.k*	LEU3kR	CTCCATATACTTCGCATTATC	This study
*LRO1*	*S.c.*, *S.u*	LRO1F	AAAGCTGGGGAGTTATTGGA	^35^
		LRO1R	TGGGTTGTTCACCCCGTATAT	
	*S.k*	LRO1kR	TTTTCTTCCTTGTACACATACG	This study
*MAG2*	*S.c.*, *S.u.*, *S.k*	MAG2F	ATGGTCGAACGGGATATGCAGAAGAAGGC	^36^
		MAG2R	AGCTCCAAAGAATAAGATACACCACATTTCAT	
*MAT*	*S.c*	MATa^Sc^	CCACATTAAAAAAGAGAAGAGC	^19^
		MATα^Sc^	TAAAATCCAAATTCACAGGATAGCGTCT	
		Scout	TATGGTTAAGATAAGAACAAAGAATG	
	*S.k*	MATa^Sk^	GTATGAAAAATCAAGCTAA	^19^
		MATα^Sk^	GTAATGGCATAGTGAAACGAATAAGT	
		Skout	GTAAATACCTCAAAGGAATTATCA	
	*S.u*	MATa^Su^ MATα^Su^	CAACGTGAATCAATCCTAA TCGAGAAAAGCATCAATAACAC	^19^
		Suout	TCACCAAATACGAAAAGTAA	
*MET2*	*S.c.*, *S.u.*, *S.k*	MET2F	CGAAAACGCTCCAAGAGCTGG	This study
		MET2R	GACCACGATATGCACCAGGCAG	
*MNT2*	*S.c.*, *S.u.*, *S.k*	MNT2F	ATACAGATCTATCTTTTTGGGAGAACTGG	^36^
		MNT2R	AGTCTCTGGCTATGCTCATAATCGTATTCCCA	
*OCA5*	*S.c.*, *S.u*	OCA5F	CGCCCTCTATCTTGTCTTTGT	^35^
		OCA5R	TGCCATCGTGAAATTTCTGC	
	*S.k*	OCA5kF	CAATACTGCTCGTTATCAC	This study
*OPY1*	*S.c.*, *S.u.*, *S.k*	OPY1F	CCGCGGACAACAGACCAYCATTAYTGGTGYGT	^36^
		OPY1R	CTCTTGAAATTTATTATCCARTCCACCATRTCYTG	
*PRP4*	*S.c.*, *S.u*	PRP4F	ACAAAATGAAAGCACCGCTGA	^35^
		PRP4R	CAAACAAGAGATCCATCGCA	
	*S.k*	PRP4kF	CTCAAGATGAAAGTACTGCCG	This study
*PTR2*	*S.c.*, *S.u*	PTR2F	TCCGCACCATTCCAAAACTA	^35^
		PTR2R	GCCAAACCAGTGAATAACCA	
	*S.k*	PTR2R	CGGAAATTGGATTCAAGTC	This study
*RDR1*	*S.c.*, *S.u.*, *S.k*	RDR1F	GGCAAATCTCCATGTGAAATG	This study
		RDR1R	AATCTCATGATGCAGGCCAA	
*SAM3*	*S.c.*, *S.u*	SAM3F	CCGCTTTGCTAATCGGTTTT	^35^
		SAM3R	TCCTTGAGCTTTCAAAGCCA	
	*S.k*	SAM3kF	GGGACAGGTCTTTTCATCGGTTTGGG	This study
*SNF1*	*S.c.*, *S.u*	SNF1F	GATTGCCGATTTTGGTTTGTC	This study
		SNF1R	TGATCCATGAAGGGTGATTG	
	*S.k*	SNFkR	CGAGCTCATCACTGACAT	This study
*STE2*	*S.c.*, *S.u*	STE2F	TTGTCATGTGGATGACATCGA	^35^
		STE2R	GGTGTGGGCAACTGATAAAA	
	*S.k*	STE2kF	AGCGATCTGTTTTATGATCC	This study
		STE2kR	TTGTTGATGCTGTCAGTTTG	
*STE6*	*S.c*	STE6cF	CATACGGAATGACTACAGGCTG	This Study
		STE6cR	CTGCTTCCGAAGGTCTGCTTGG	
	*S.k*	STE6kF	CGACGACTGAGGAACAACACC	This study
		STE6kR	CCATCAAGTGTTTACAGGCT	
	*S.u*	STE6uF	TGAGCATACGCAATGACTAC	This study
		STE6uR	GATGCTCTCTAGTATTCGAGGCA	
*TDA1*	*S.c.*, *S.u.*, *Sk*	TDA1F	ACCACAACTCCTTGGGCGAT	^35^
		TDA1R	TCAACGTAAAGGTCAGGCAA	
*UBP7*	*S.c.*, *S.u.*, *S.k*	UBP7F	CCTCTTAGGTGGGTATGAAAAATGGAAAAAAAC	^36^
		UBP7R	CCATTAACAATTACGTTTTTATCAAACCAGTG	
*UGA3*	*S.c.*, *S.u*	UGA3F	CGCCCATGAACCAGAACTACT	^35^
		UGA3R	GCCATAAGCGAAGGTTGTAA	
	*S.k*	UGA3kF	GCGTGGAGAAGCTGAAACTG	This study
		UGA3kR	GGGGAAGATTATCCTCTCCTCTAGG	

^1^*S*.*c. S. cerevisiae*, *S.k*. *S. kudriavzevii*, *S.u. S. uvarum.*

### mtDNA extraction and RFLP

Mitochondrial DNA was prepared from exponential-phase YEL cultures with the method described by Nguyen et al.^28^ and digested with *Mbo*I. The fragments were separated by gel electrophoresis in 0.7% agarose, 0.5 × TBE.

### Mating and spore viability tests

Mating activity was tested in exponential-phase mixed cultures as described previously^19^. Briefly, equal volumes of overnight cultures of the strains grown in YEL were mixed, centrifuged and then 10 μl of the wet pellet was dropped on YEA. After incubation at room temperature for 4–6 h, samples were taken and examined microscopically. The testers of mating competences were the parental strains of the hybrids.

Spore viability was examined by tetrad analysis. Samples of cultures grown on the sporulation medium SPA at room temperature for 5 days were suspended in Zymolyase-T20 (0.05 mg ml^−1^) solution. After incubation at 37 °C for 20 min, aliquots were streaked on YEA plates, and four-spored asci were pulled out from the streaks with a Carl Zeiss 2588 micromanipulator. The asci were dissected with the micromanipulator and the free spores were separated from each other on the plate to let the viable spores form individual colonies.

### Physiological tests

Strains were tested for the utilisation of sugars as carbon sources in Durham tubes filled with YEL in which glucose was replaced with different sugars. The sensitivity of the strains to higher temperatures was compared by culturing them on YEA plates at 25 °C and 35 °C for 3 days. Their ability to flocculate was examined by culturing them in YEL on an orbital shaker at room temperature for 2 days. To visualise the aggregates, the cultures were poured into glass Petri dishes and photographed on dark background.

## Results

### Construction of sterile allodiploid and allotetraploid two-species and three-species hybrids

By mating double auxotrophic heterothallic *S. kudriavzevii* and *S. uvarum* strains, sterile ura^-^ kudvarum hybrids were produced. Despite their sterility, the ura^-^ kudvarum hybrids formed prototrophic three-species cekudvarum hybrids at low frequency with the *S. cerevisiae* strain having complementary auxotrophy. The cekudvarum cells were also sterile. One kudvarum strain (II/6) and its 5 cekudvarum hybrids (II/6.1 to II/6.5) were chosen for further examination. II/6, II/6.1 and II/6.2 were used in a parallel project for the investigation of the role of the *MAT* locus in the yeast-specific second sterility barrier^19^ but their genomes were not examined in detail.

The genome size of the hybrids and their parental strains was compared by flow cytometry analysis (Fig. 1). The fluorescence peaks of the heterothallic parental strains 10–1651 *S. uvarum* and 10–1653 *S. kudriavzevii* had identical positions and could be attributed to cells being in G1 (1C amount of DNA) and G2 (2C amount of DNA) phases of the cell cycle. The positions of the *S. cerevisiae* culture indicated that its cells had 2C and 4C amount of DNA in the G1 and G2 phases. The increased genome size can be attributed to the instability of the heterothallism of this strain. It forms asci on the sporulation medium and rarely also on YEA. Sporulation indicates that it has become homothallic and its cells are diploid. The three-species hybrids which we produced in our previous study and analyse here might have arisen by rare mating between sterile allodiploid kudvarum cells and sterile diploid *S. cerevisiae* cells (allotetraploid three-species hybrid) or by “half-rare” mating between sterile kudvarum cells and fertile haploid *S. cerevisiae* ascospores (allotriploid three-species hybrid). However, the flow cytometry analysis measured tetraploid genomes (a peak located in a position corresponding to the 4C peak of the kudvarum parent and a peak behind it). Thus, the three-species cekudvarum hybrids had tetraploid amount of DNA. This result makes it unlikely that the hybrids were formed by “half-rare” mating.

### The hybrids have alloeuploid karyotypes

**Figure 2:**
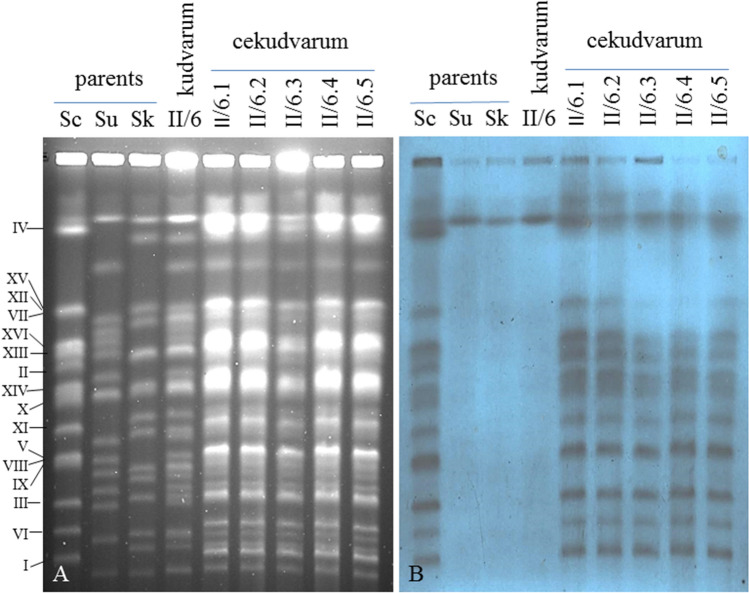
Karyotyping. (**A**) Separation of chromosomes. (**B**) Southern hybridisation with *S. cerevisiae* Y’ telomeric sequence. Sc: 10–170 *S. cerevisiae*. Su: 10–1651 *S. uvarum*. Sk: 1653 *S. kudriavzevii*. Designation/numbering of *S. cerevisiae* chromosomes is shown on the left side.

Measuring of the DNA content of cells by flow cytometry gives information about the ploidy, but provides no insight in the composition of the genome. It is not suitable for the investigation of the contribution of the parental genomes to the hybrid genome. The increased size of the latter can be due to the presences of complete parental subgenomes or to partially incomplete and partially duplicated subgenomes. Since the number of the chromosomes is identical in the three species used in this study but many of them differ in size^24,29^, the origin of most chromosomes of a hybrid can be inferred from their size. Therefore, we compared the karyotypes of the hybrids with those of the parental strains by pulsed-field gel electrophoresis. The karyotype of the two-species hybrid (II/6) shown in Fig. 2A contained all chromosomal bands of both parental strains. The number of bands further increased in the three-species karyotypes but certain chromosomes were not separated clearly. Neither the extension of the run-time of the electrophoresis nor the changes of the running parameters could separate them unambiguously. Although it could logically be supposed that the drastic increase in the number of bands was due to *S. cerevisiae* chromosomes, we wanted to prove this fact experimentally. Therefore we probed the gel with labelled *S. cerevisiae*-specific Y’ telomeric sequences which only bind to the *S. cerevisiae* chromosomes^28^. As shown in Fig. 2B, all chromosomes of the *S. cerevisiae* parent and their size equivalents in the three-species genomes bound the probe. The single positive band in the other parental karyotypes can be attributed to non-specific binding. From the results of the flow cytometry and karyotype analyses it can be concluded that both types of hybrids had alloploid genomes.

### PCR–RFLP analysis of marker genes verifies the euploidy of hybrids

**Figure 3:**
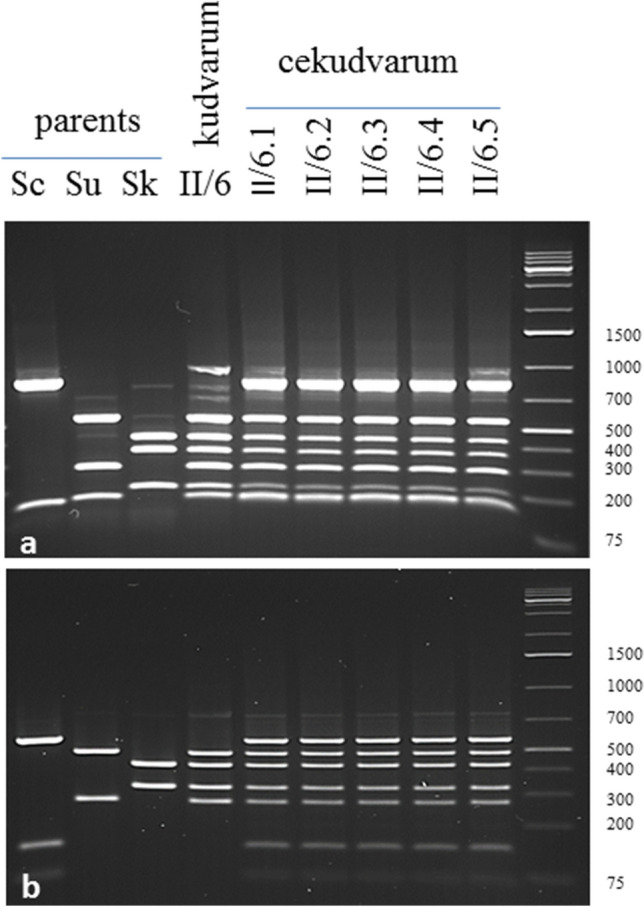
Examples of PCR–RFLP restriction patterns. (**a**) *MNT2* digested with *Msp*I. (**b**) *RDR1* digested with *Hae*III. Sc: 10–170 *S. cerevisiae*. Su: 10–1651 *S. uvarum*. Sk: 1653 *S. kudriavzevii*. *MNT2* is located on Chr VII^Sc^, Chr 7^Sk^ and Chr 5^Su^. *RDR1* is located on Chr XV^Sc^, Chr 15^Sk^ and Chr 8^Su^. Size ladder on the right side.

To confirm that the hybrids had euploid genomes, we tested them for the presence of parental orthologues of 34 “genetic markers” (genes and loci) that covered the entire chromosomal sets of the parental strains. The orthologues of the markers could be differentiated by PCR–RFLP due to their different restriction patterns (Supplementary Table S1 and examples in Fig. 3 and Supplementary Fig. 2S). Since the genomes of the three species are not entirely syntenic, 11 markers were located on different (non-homeologous) chromosomes (marked with grey in Table 2) in their genomes. Therefore at least two markers were chosen for each of the 16 chromosomes, in most cases from different arms. In the case of three markers *GND1*, *CYR1* and *MET2* (located on Chr VII, X and XIV of *S. cerevisiae*, respectively) the restriction patterns did not differ sufficiently for distinguishing all three orthologous (Table S1). The bands of the *S. kudriavzevii* pattern of *OPY1* (Chr II in *S. cerevisiae*) were not visible in the hybrids. Since other markers of these chromosomes showed different parental patterns, these chromosomes could also be detected. The PCR–RFLP analysis identified all *S. kudriavzevii* and *S. uvarum* chromosomes in the two-species kudvarum hybrid II/6. The three-species cekudvarum hybrids also had all *S. cerevisiae* chromosomes. Taking all PCR–RFLP results together, it can be concluded that both the two-species and the three-species hybrids had complete sets of parental chromosomes.

### The mitochondrial genome is inherited uniparentally

**Figure 4:**
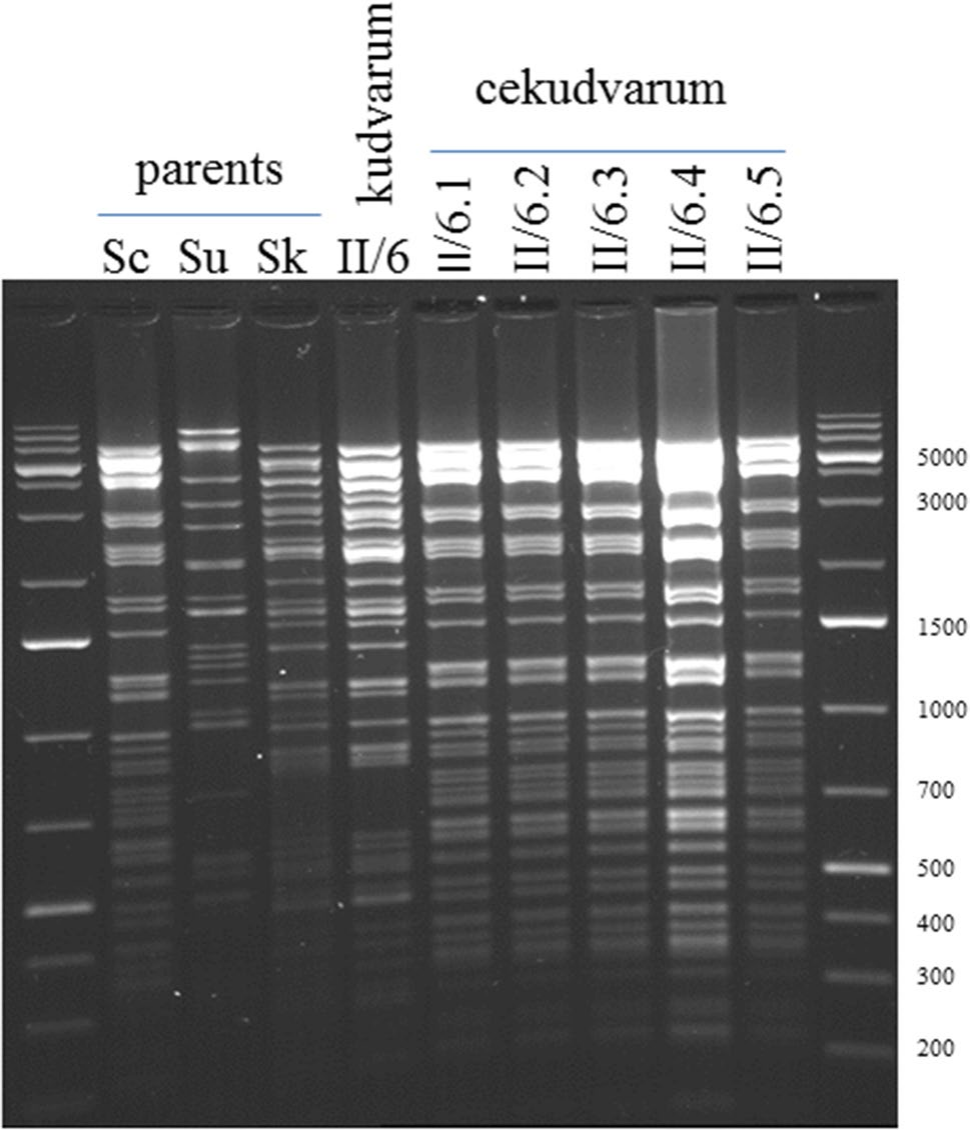
RFLP patterns of the mitochondrial DNA digested with *Mbo*I. Sc: 10–170 *S. cerevisiae*. Su: 10–1651 *S. uvarum*. Sk: 1653 *S. kudriavzevii*. Size ladder on the right side.

Digestion of the isolated mitochondrial DNA with *Mbo*I generated different band patterns for the parental strains (Fig. 4). The pattern of the two-species hybrid II/6 was identical with that of the *S. kudriavzevii* parent. The 5 three-species hybrids had identical mitochondrial genomes whose *Mbo*I patterns were indistiguishable from that of the *S. cerevisiae* parent. Thus, the hybrids were homoplasmic and the mitochondrial genomes were inherited uniparentally.

### Dominant/recessive relationships in the determination of phenotypic traits

Since the species used for hybridisation differ in certain taxonomically relevant phenotypic traits, we tested the hybrids for these properties. The growth of *S. uvarum* is inhibited by temperatures above 35 °C, whereas the other species can grow at these temperatures. Neither the kudvarum nor the cekudvarum hybrids were sensitive to 35 °C, so the temperature sensitivity of *S. uvarum* is recessive (Supplementary Fig. 3S).

**Figure 5:**
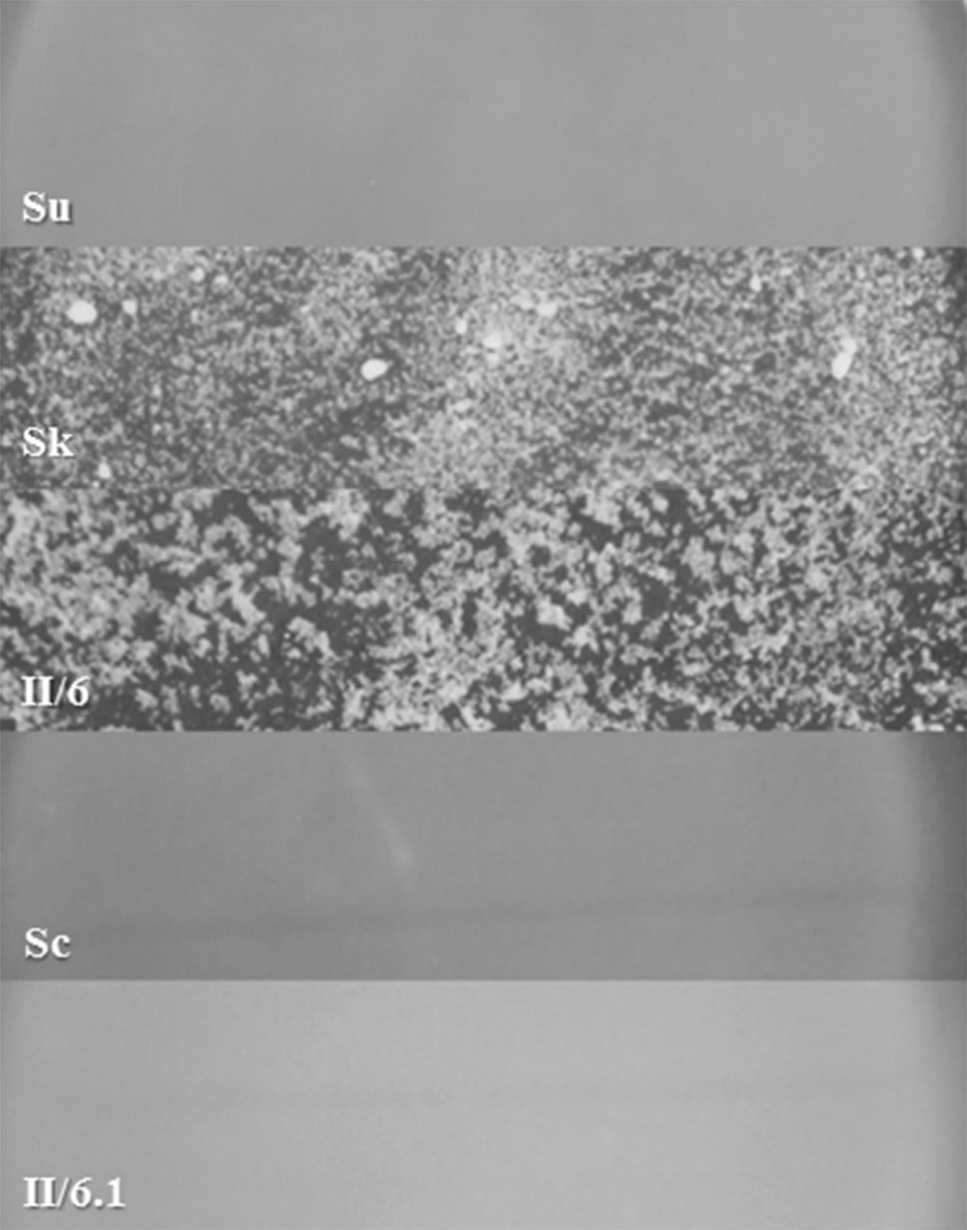
Flocculation of cells. Su: 10–1651 *S. uvarum*. Sk: 1653 *S. kudriavzevii*. II/6 two-species kudvarum hybrid. Sc: 10–170 *S. cerevisiae*. II/6.1: three-species cekudvarum hybrid.

*S. uvarum* can utilise melibiose as a carbon source whereas the other species are mel^−^. Both types of hybrids grew in the medium in which glucose was replaced with melibiose and also could ferment it. Thus, this trait of *S. uvarum* was dominant. *S. uvarum* and *S. kudriavzevii* also differ in maltose and galactose utilisation (*S. uvarum* is mal^+^ and gal^+^) and flocculation of cells (*S. kudriavzevii* is highly flocculant). All hybrids were able to utilise both carbon sources, indicating that these traits are also determined by dominant alleles. The genetic determination of flocculation appears to be more complex. As shown in Fig. 5, II/6 flocculated like the *S. kudriavzevii* parental strain but the cekuvarum hybrids did not flocculate.

## Discussion

In a previous study we created two-species kudvarum and three-species cekudvarum hybrids to investigate the role of the *MAT* locus in the postzygotic sterility barriers that biologically isolate the *Saccharomyces* species from each other^19^. Here additional hybrids were produced and the genome structures of selected representatives were investigated. As expected on the basis of numerous previous observations (reviewed e.g. in Reference^6^), the two-species hybrids were sterile. In many plants, the sterility of interspecies hybrids can be overcome by genome duplication (e.g.^12^). *Saccharomyces* allodiploid hybrids can also duplicate their genomes but the duplication does not restore fertility because the yeast allotetraploids do not form functional (mating-competent) gametes. However, occasional imprecise partitioning of chromosomes during allotetraploid meiosis can result in mating-competent spores. The spore receiving only one *MAT*-carrying chromosome (loss of *MAT* heterozygosity) can conjugate with other spores or cells^14,19^. The regained fertility allows hybridisation with a third species but the hybrids will not have complete parental genomes because the lost chromosome(s) will be missing^21,22^. Since we wanted to create three-species hybrids possessing euploid genomes (complete parental subgenomes), we opted for a different hybridisation strategy. We made use of the rarely occurring “escape” from the repression of the mating programme by the *MAT* heterozygosity “are mating”^23^). Although rare mating was originally observed in *S. cerevisiae* autodiploids, we found in this study that mating-competent cells also occur in kudvarum allodiploid cultures that can mate with *S. cerevisiae* cells to form three-species cekudvarum hybrids.

The flow cytometry analysis determined 2C and 4C amounts of DNA in the kudvarum and cekudvarum hybrids, respectively. Since the *S. kudriavzevii* and *S. uvarum* strains were stable heterothallic haploids and the *S. cerevisiae* was diploid, we inferred from the flow cytometry results that the kudvarum hybrids had allodiploid n^Sk^n^Su^ genomes and the cekudvarum hybrids had allotetraploid 2n^Sc^n^Sk^ n^Su^ genomes. In the electrophoretic karyotypes, the hybrids had equivalents of all chromosomal bands of the parents.

However, neither flow cytometry analysis nor karyotyping can unambiguously prove that the hybrids have complete (euploid) subgenomes. The FACS analysis is not sufficiently sensitive to detect differences in DNA content arising from loss or duplication of single chromosomes, and karyotyping cannot separate chromosomes similar in size. To identify each chromosome individually, we tested the hybrids for the presence of orthologues of a group of selected genes as chromosome-specific molecular markers that covered all chromosomes of all parental strains. The RFLP analysis of these markers identified complete sets of *S. kudriavzevii* and *S. uvarum* chromosomes in the two-species kudvarum hybrids, and the three-species cekudvarum hybrids also had all *S. cerevisiae* chromosomes. Therefore, both types of hybrids had euploid genomes.

Neither the kudvarum nor the cekudvarum hybrids formed viable spores. The failure of interspecies allodiploid hybrids to produce viable gametes can be attributed to the failure of the allosyndetic (homeologous) chromosomes of their subgenomes to pair during prophase I of meiosis (e.g.^8–11^). Even if the homeologous chromosomes are syntenic enough for aligning with each other, their sequence differences prevent them from efficient DNA strand exchange necessary for pairing up in Prophase I^11^. Since both types of hybrids had single copies of *S. kudriavzevii* and *S. uvarum* chromosomes, normal meiosis could not take place and viable gametes could not be produced. The presence of two sets of *S. cerevisiae* chromosomes did not improve the situation despite the possibility of normal pairing within the *S. cerevisiae* subgenome. Previous studies have shown that when the alloploid (e.g. allotetraploid) hybrid had eudiploid subgenomes, the chromosomes paired preferentially with their homologues within the autodiploid subgenomes. This mode of meiosis, referred to as autodiploidised allopolyploid meiosis, produces viable spores^15^. The cekudvarum hybrids could not form viable spores because only one of the subgenomes was autodiploid.

Since no mitochondrial markers were used in the construction of hybrids, the transfer of the mitochondria from the parental cells to the hybrids did not take place under selection pressure. In such circumstances, the mitochondria of both mating partners can be transmitted into the zygote. However, heteroplasmic interspecies hybrids were rarely observed when different *Saccharomyces* species were hybridised in previous studies. The hybrids usually had parental mitotypes or, less frequently, recombinant mitotypes (e.g.^16,30–34^). In this study both types of hybrids were homoplasmic. The two-species kudvarum hybrids received their mtDNA from *S. kudriavzevii*. This was then replaced with the mtDNA of *S. cerevisiae* in the three-species cekudvarum hybrids. In both cases the mitochondrial genome was inherited uniparentally. In previous studies, we also observed uniparental inheritance of *S. cerevisiae* mitochondrial genome in cevarum (*S. cerevisiae* x *S. uvarum*) hybrids^16,24^.

Three-species hybrids provide possibilities to study the interactions of three orthologues (alleles) of genes within one strain. In a previous paper we found that the genes of the *MAT* loci and the *HO* genes of three subgenomes cooperated in the hybrids as efficiently as their counterparts in the parental strains^19^. Here we show that the temperature sensitivity of *S. uvarum* is recessive both in the two-species and in the three-species hybrids, whereas the ability of this species to utilise galactose, maltose and mellibiose as carbon sources is dominant. The relationships of the determinants of flocculation appear to be more complex: this trait characteristic of the *S. kudvarum* cells was dominant in kudvarum but recessive in the cekudvarum hybrids.

The results presented in this study demonstrate that three-species euploid hybrids can be constructed by making use of natural mating processes and complementation of auxotrophic phenotypes without the application of genetic engineering. These hybrids allow the investigation of interactions of complete gene pools of three species, subsets of genes involved in complex physiological properties and individual groups of orthologues. Being non-GMOs, these hybrids and their segregants formed by postzygotic evolution of their genomes (e.g. by GARMi and GARMe) can be exploited in biotechnological processes even in countries whose legislations restrict or prohibit the use of genetically modified organisms.

## Supplementary Information

Supplementary Figure S1.

Supplementary Figure S2.

Supplementary Figure S3.

Supplementary Information.

Supplementary Table S1.

## Data Availability

All data generated or analysed during this study are included in this published article and its supplementary information files.
