# Supplementary material for: Synthetic two-species allodiploid and three-species allotetraploid Saccharomyces hybrids with euploid (complete) parental subgenomes
Source: Sci Rep. 2023 Jan 20;13:1112. doi: 10.1038/s41598-023-27693-2 (PMC9860037; doi:10.1038/s41598-023-27693-2)
Supplement: Supplementary file 1 — Supplementary Figure S1. [file 41598_2023_27693_MOESM1_ESM.pdf]

Synthetic two-species allodiploid and three-species allotetraploid *Saccharomyces* hybrids with euploid (complete) parental subgenomes

Zsuzsa Antunovics, Adrienn Szabo, Lina Heistinger, Diethard Mattanovich & Matthias Sipiczki

re 1S. Location of marker genes on the chromosomes of the parental strains.

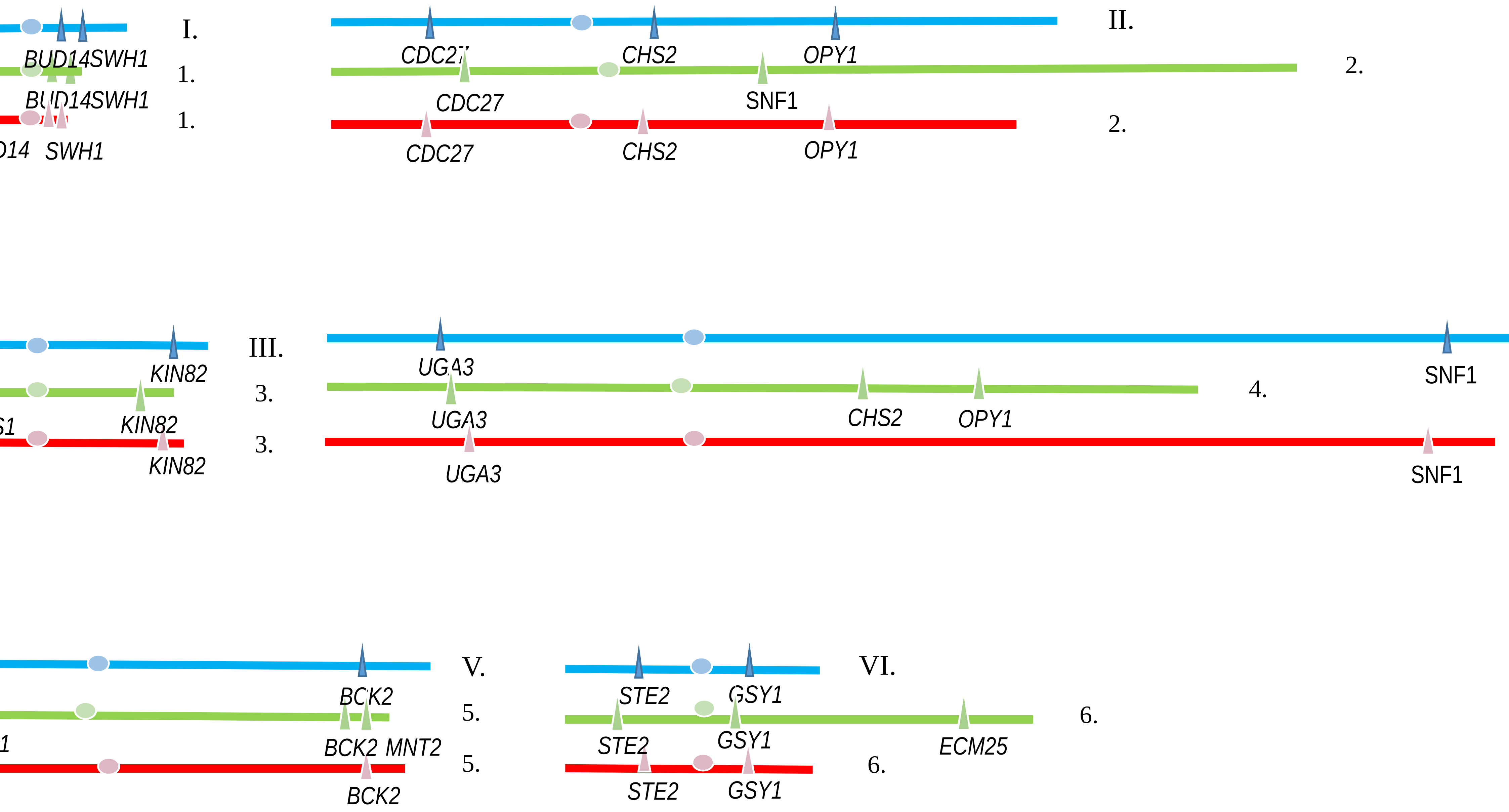

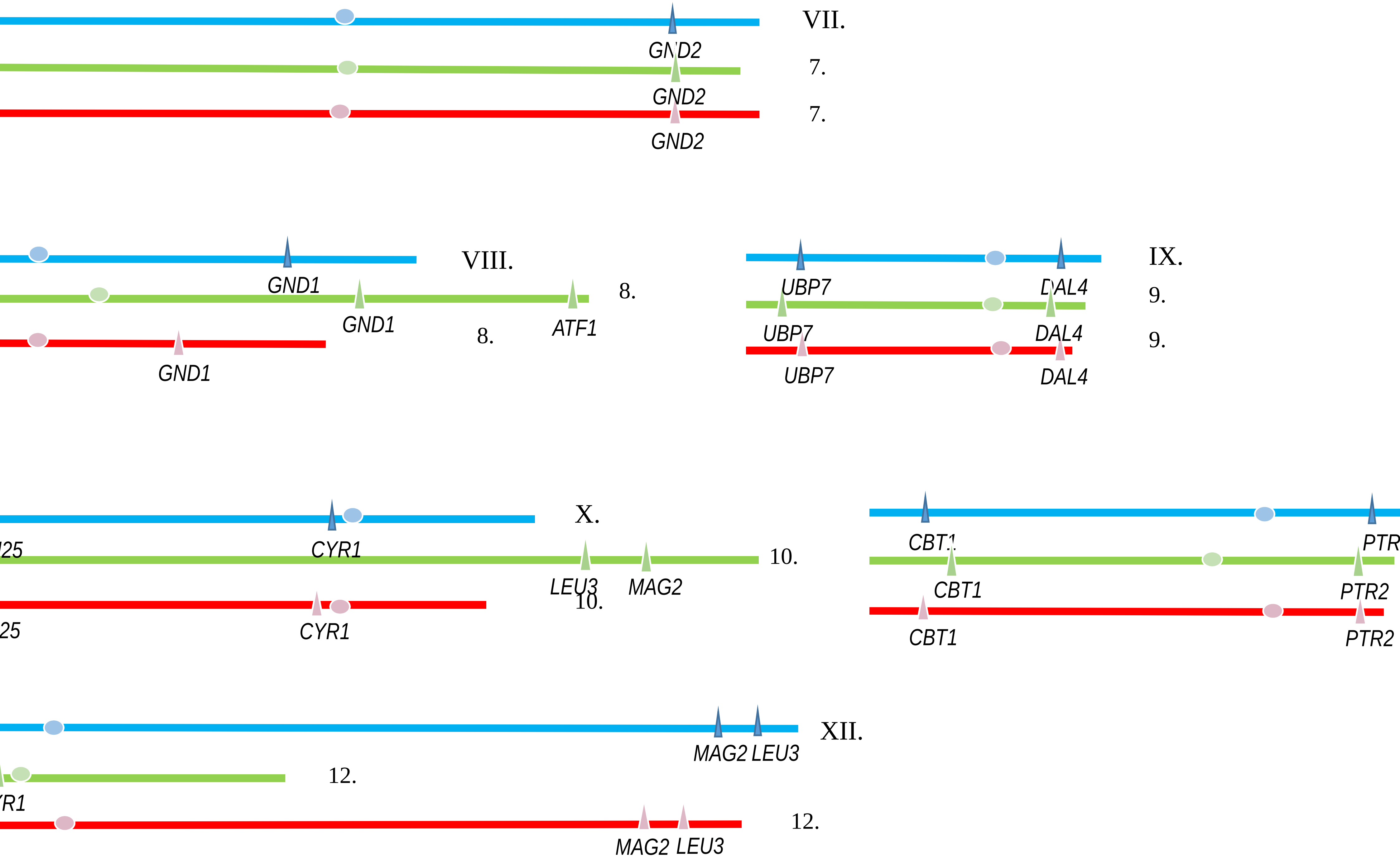
VII.
GND2
7.
GND2
7.
GND2
VIII.
GND1
8.
GND1
8.
ATF1
IX.
UBP7
DAL4
9.
UBP7
DAL4
9.
UBP7
DAL4
X.
CYR1
10.
LEU3
MAG2
10.
CYR1
CBT1
PTR2
CBT1
PTR2
XII.
MAG2 LEU3
12.
12.
MAG2 LEU3

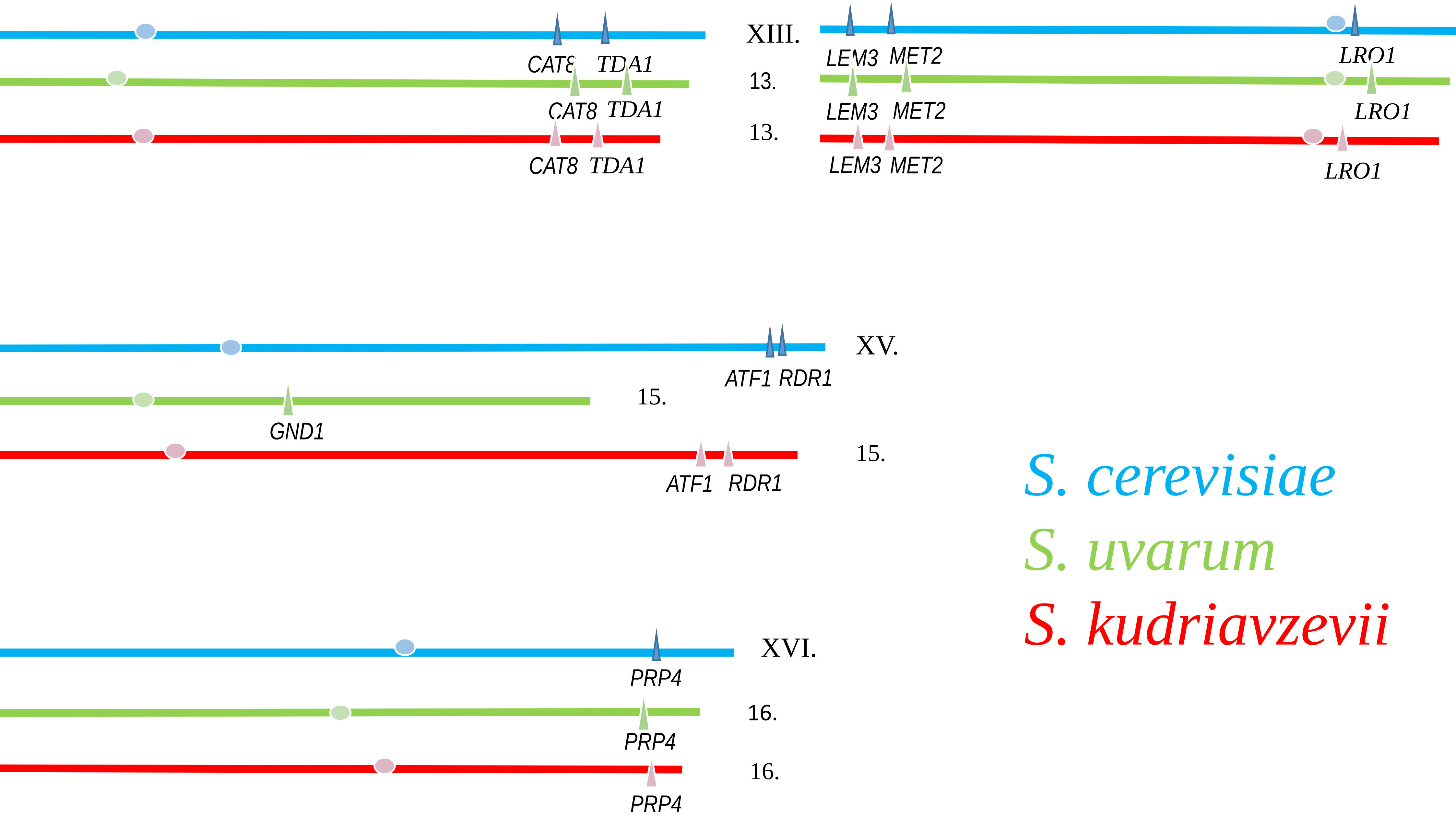

XIII.
13.
13.
CAT8 TDA1
CAT8 TDA1
CAT8 TDA1
LEM3 MET2
LEM3 MET2
LEM3 MET2
LRO1
LRO1
LRO1
XV.
15.
15.
ATF1 RDR1
GND1
ATF1 RDR1
XVI.
16.
16.
PRP4
PRP4
PRP4
S. cerevisiae
S. uvarum
S. kudriavzevii
