# Supplementary material for: Synthetic two-species allodiploid and three-species allotetraploid Saccharomyces hybrids with euploid (complete) parental subgenomes
Source: Sci Rep. 2023 Jan 20;13:1112. doi: 10.1038/s41598-023-27693-2 (PMC9860037; doi:10.1038/s41598-023-27693-2)
Supplement: Supplementary file 2 — Supplementary Figure S2. [file 41598_2023_27693_MOESM2_ESM.pdf]

Synthetic two-species allodiploid and three-species allotetraploid *Saccharomyces* hybrids with euploid (complete) parental subgenomes

Zsuzsa Antunovics, Adrienn Szabo, Lina Heistinger, Diethard Mattanovich & Matthias Sipiczki

Figure 2S. Detection of parental chromosomes by PCR-RFLP of chromosomal markers. Only examples are shown.

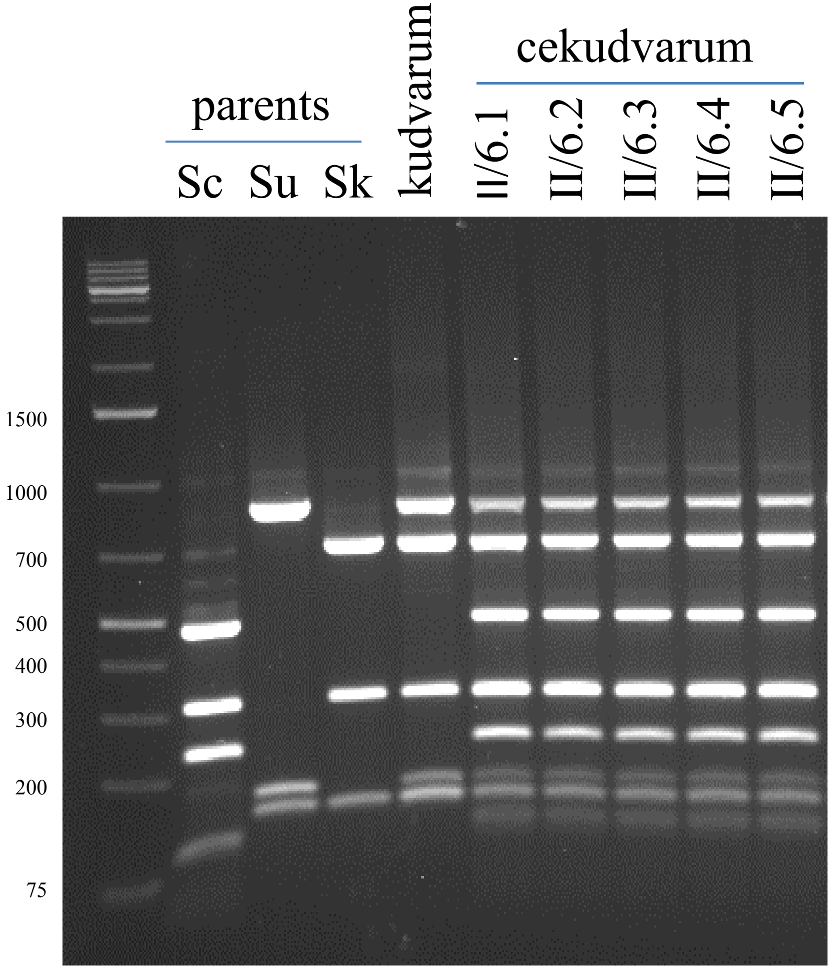

Chromosome I: *BUD14* digested with *Msp*I

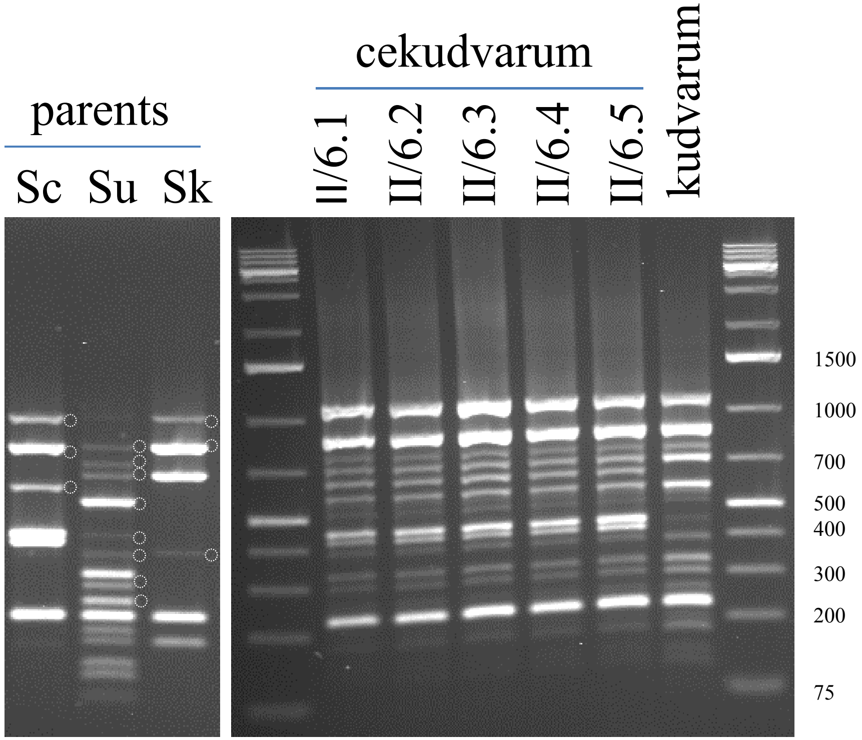

Chromosome II: *CHS2* partially digested with *Taq*I. Digestion was interrupted before completion to increase the number of fragments. ◌: incompletely digested fragments.

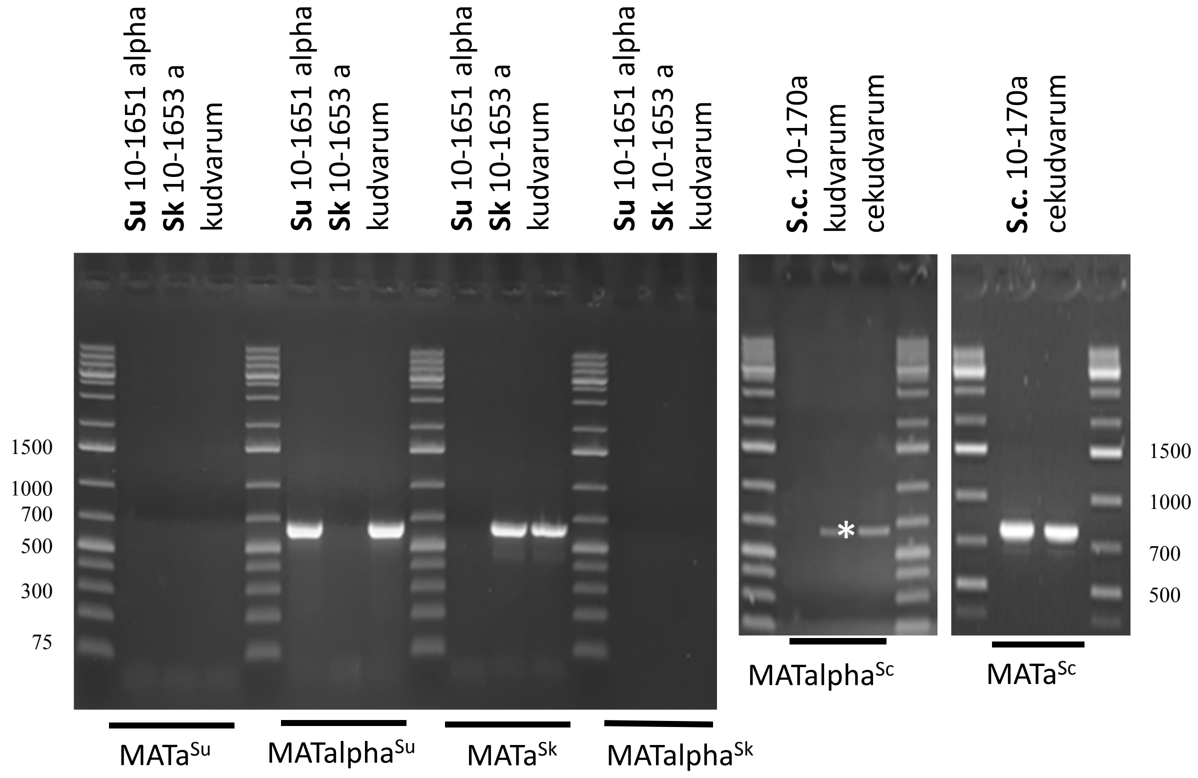

Chromosome III: *MAT* amplified with species-specific primer pairs.
*: the *S. cerevisiae MATalpha*^{Sc} primers also amplify the *S. kudriavzevii* gene with low efficiency.

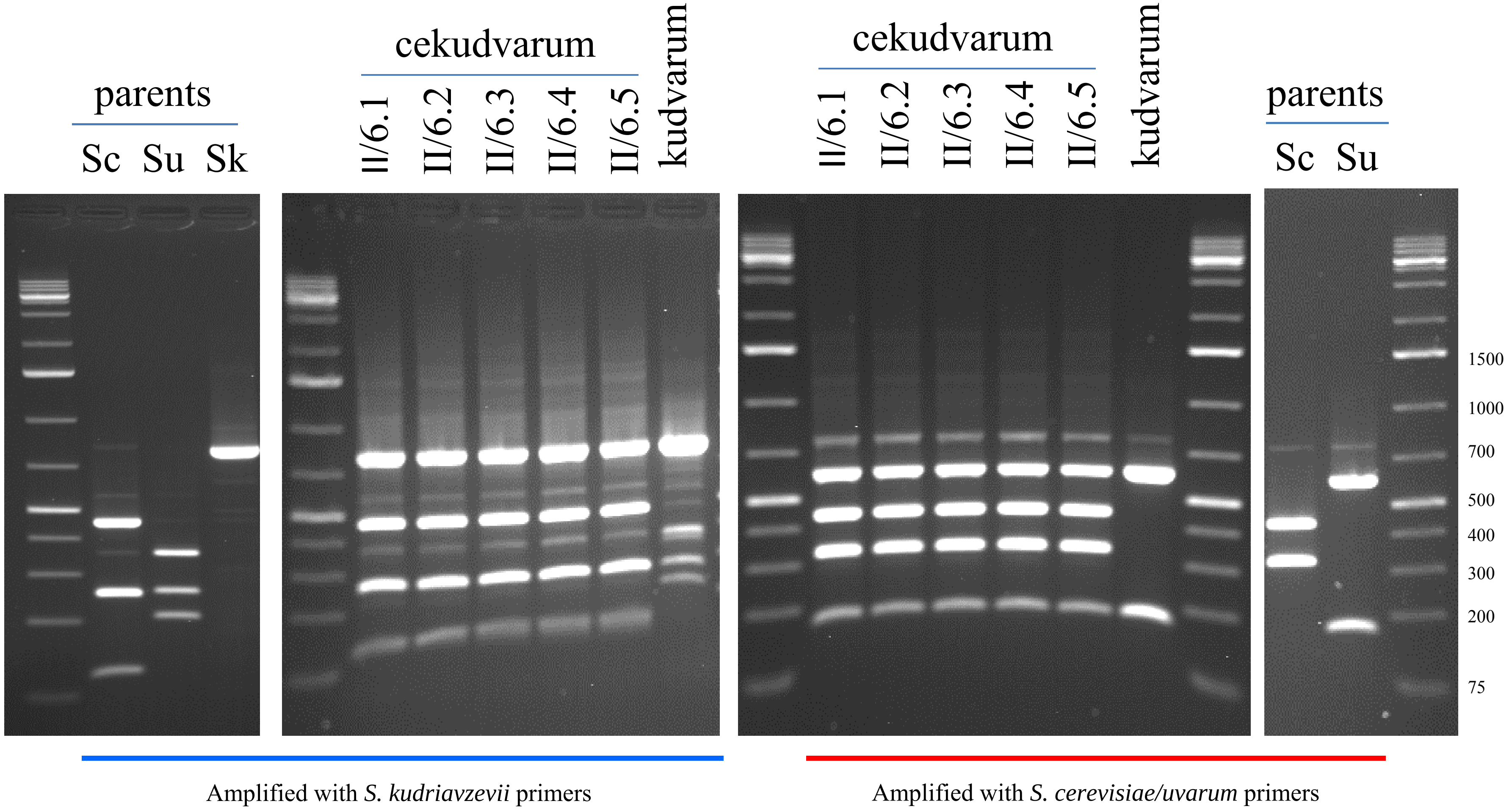

Chromosome IV: *UGA3* digested with *Rsa*I. The *S. kudriavzevii* primers amplify *UGA3* from all parental strains.

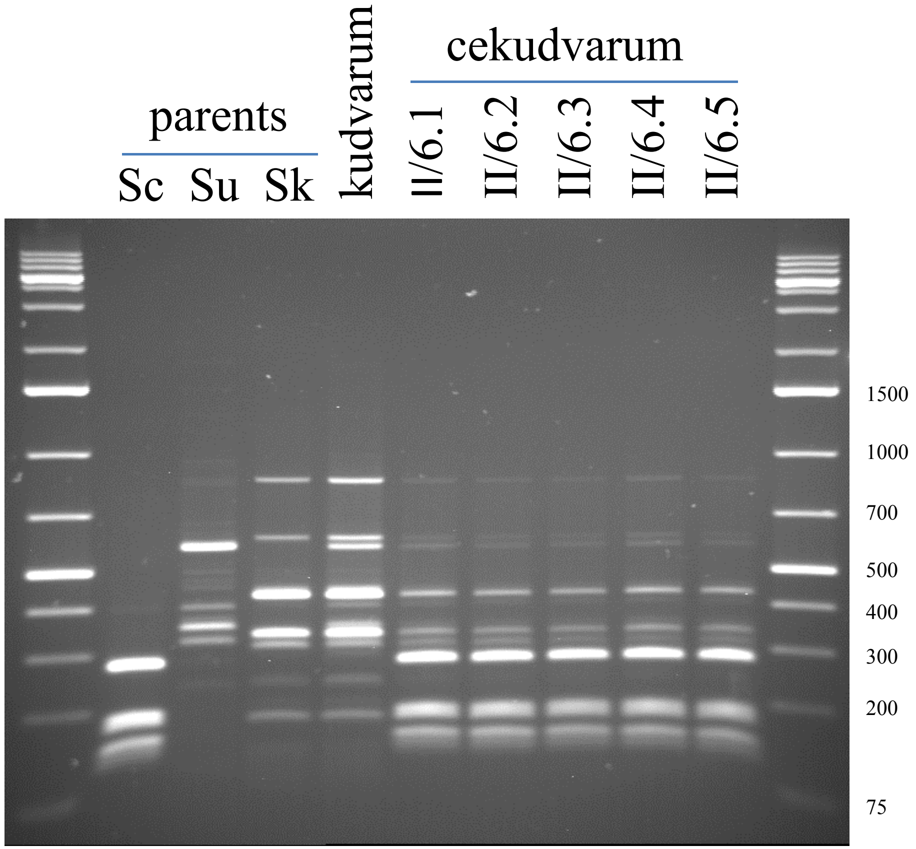

Chromosome V: *BCK2* partially digested with *Rsa*I

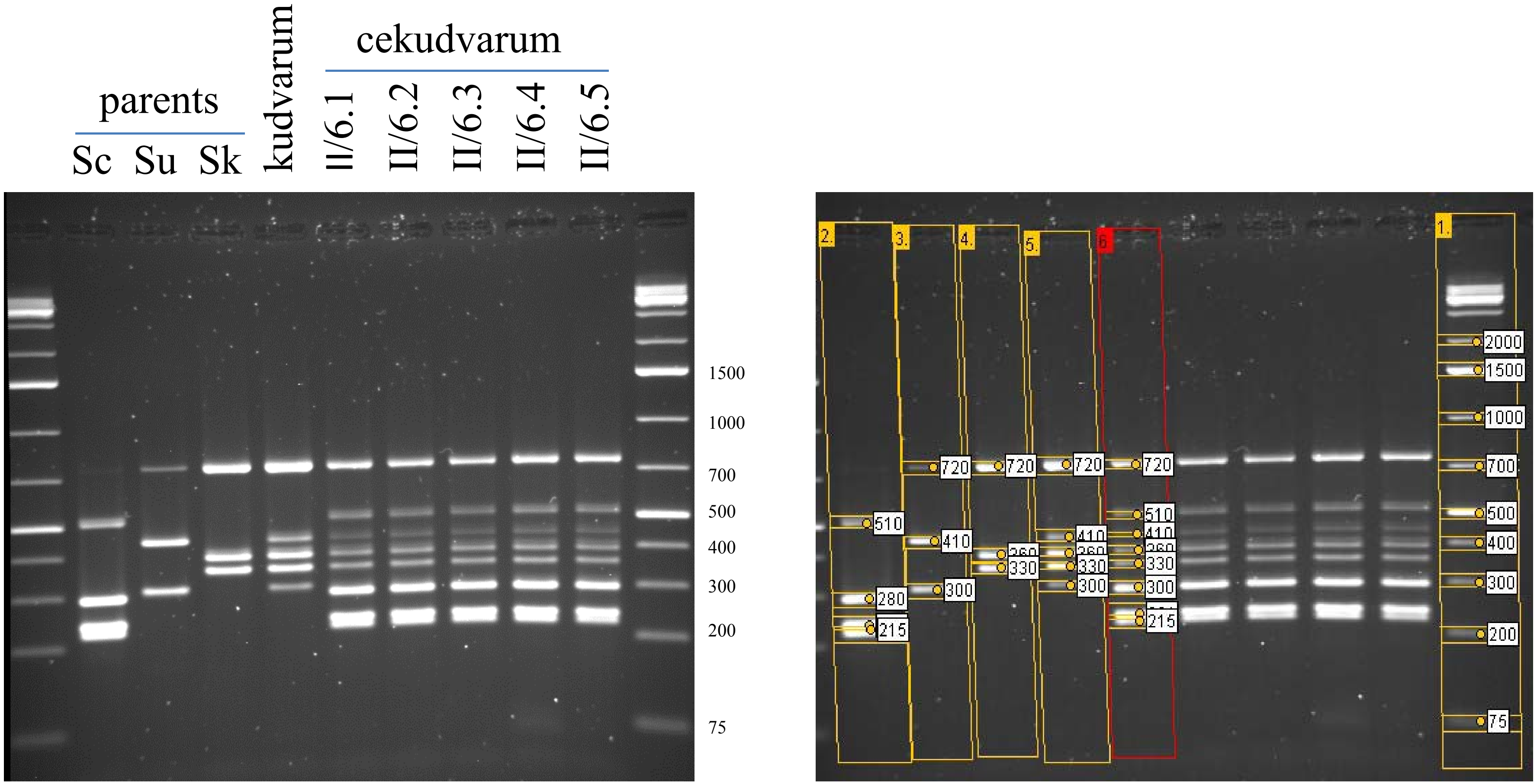

Chromosome VI: *GSY1* partially digested with *Taq*I. The bands slightly larger than 700 bp correspond to undigested amplicons. The results of fragment-size determination by gel analysis are also shown

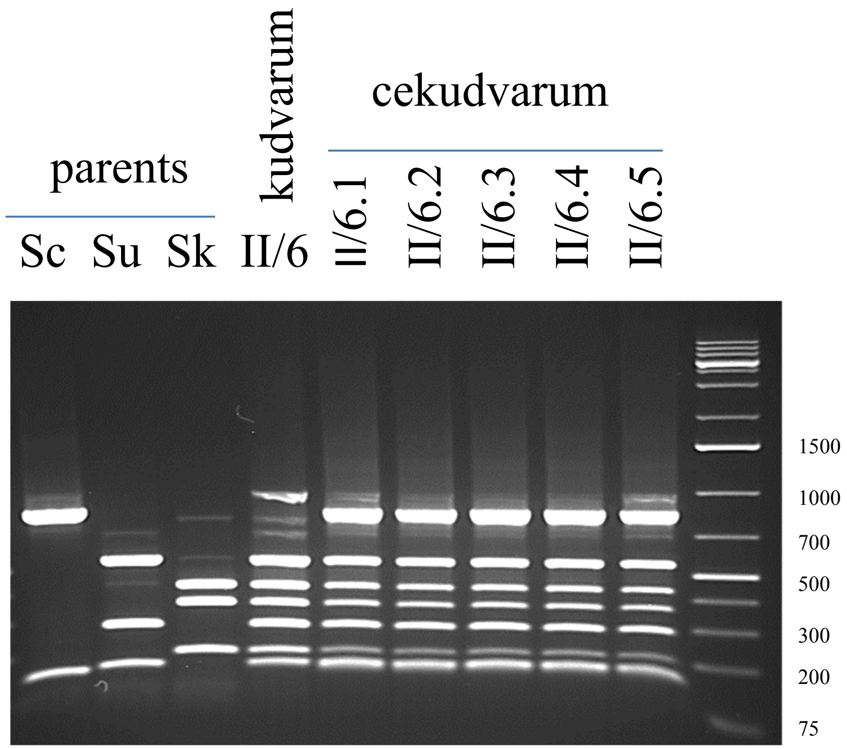

Chromosome VII: *MNT2* digested with *Msp*I

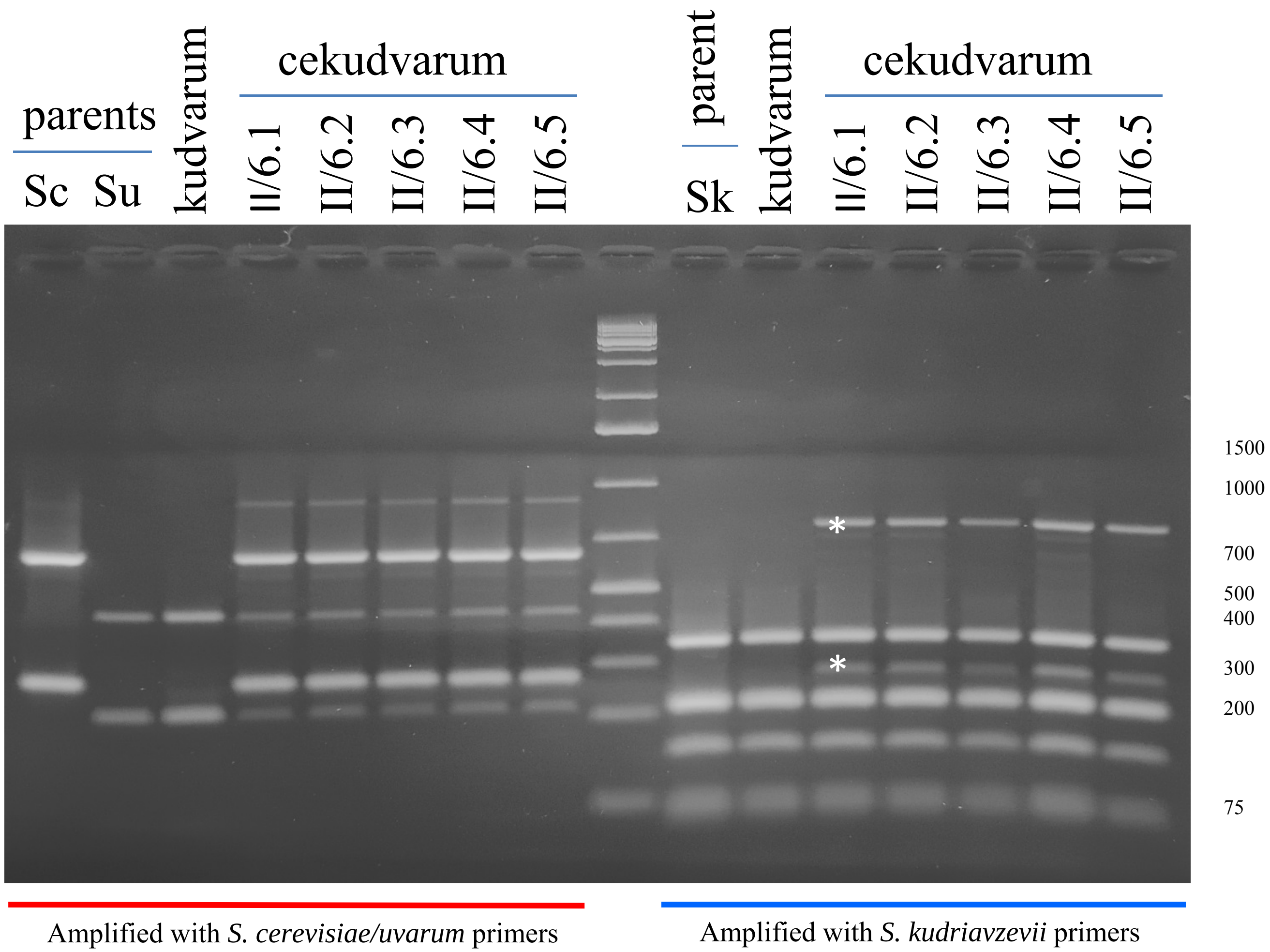

Chromosome VIII: *GND1* digested with *Hinf*I. *: fragments of *S. cerevisiae GND1*; the *S. kudriavzevii* primers also aplify the *S. cerevisiae* gene with low efficiency.

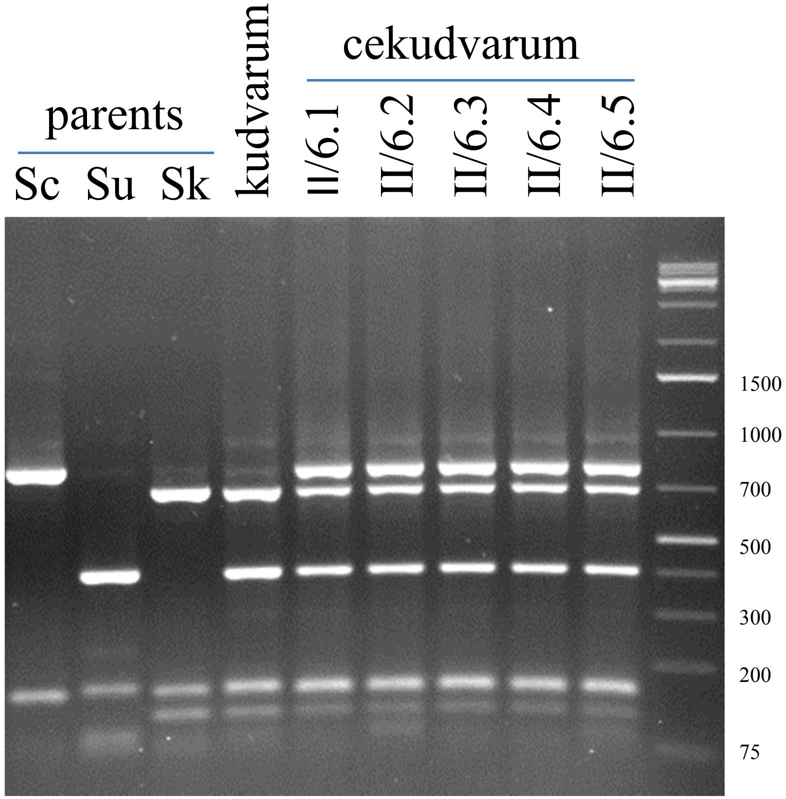

Chromosome IX: *UBP7* digested with *Hinf*I

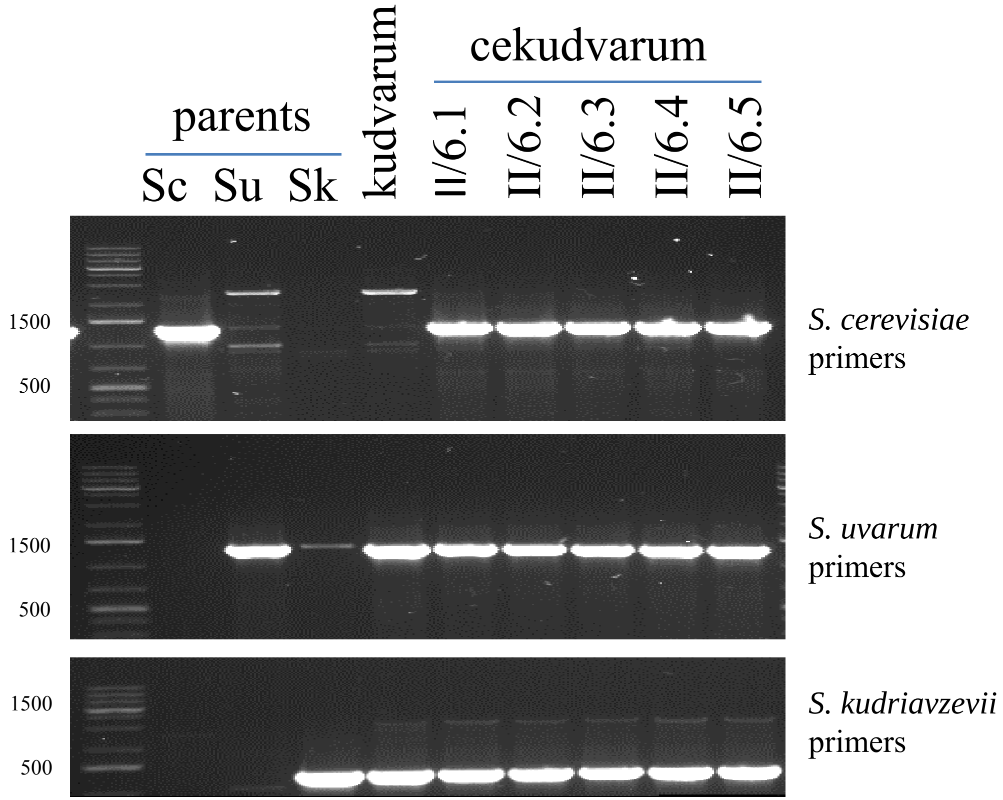

Chromosome X: *CYR1* amplified with species-specific primer pairs

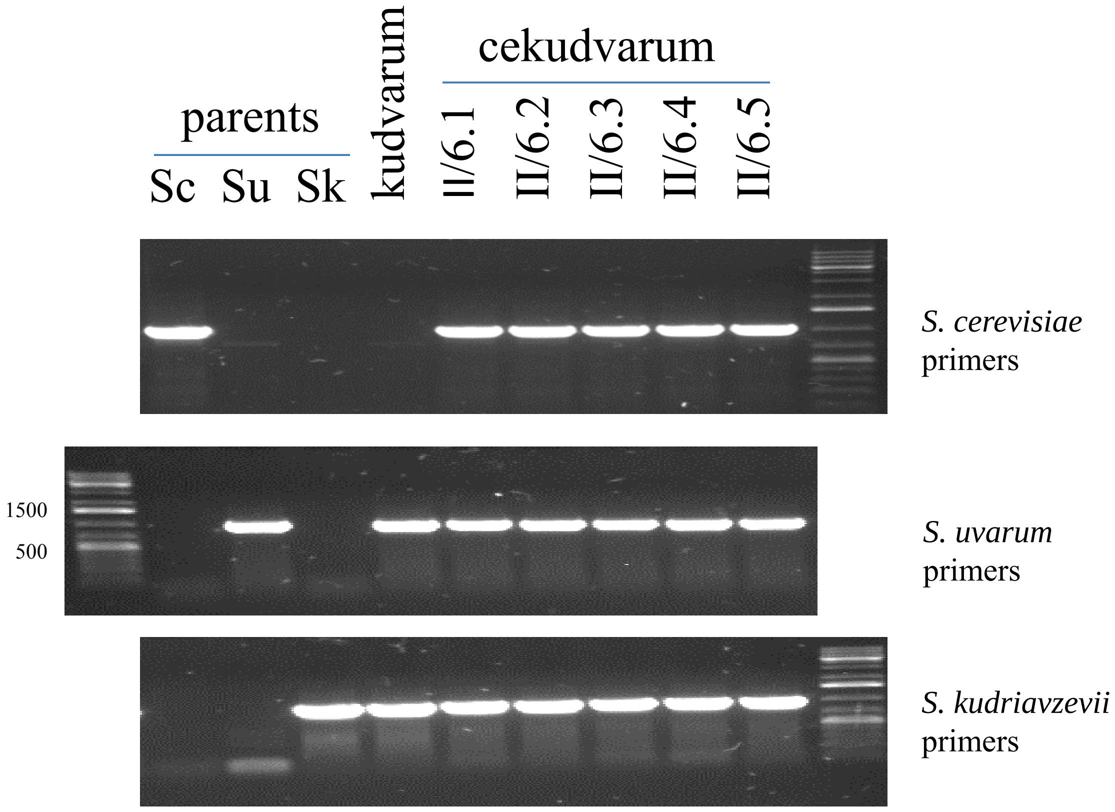

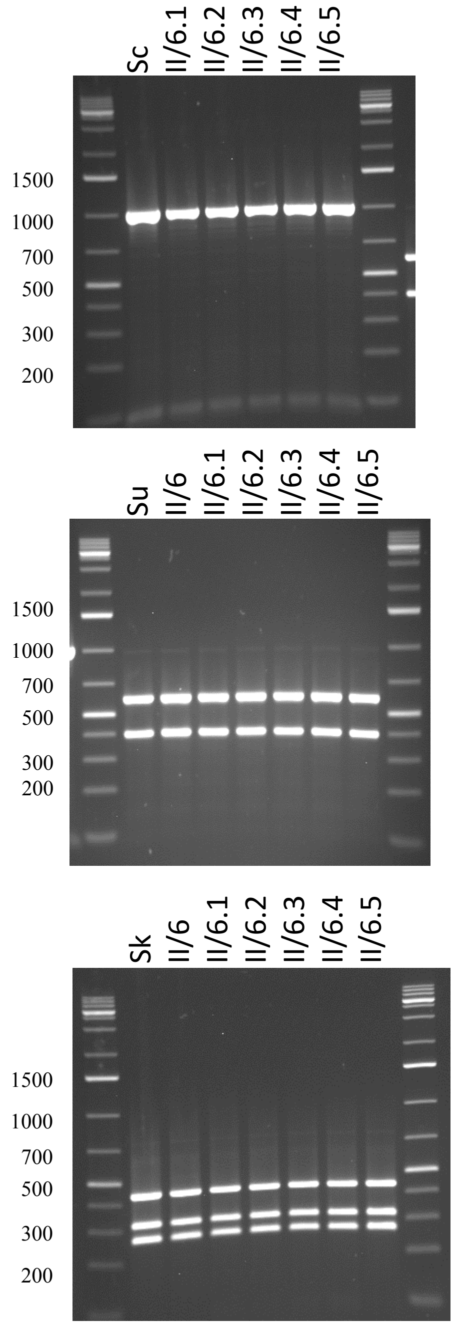

Chromosome XI: *STE6* amplified with species-specific primer pairs and digested with *Hae*III

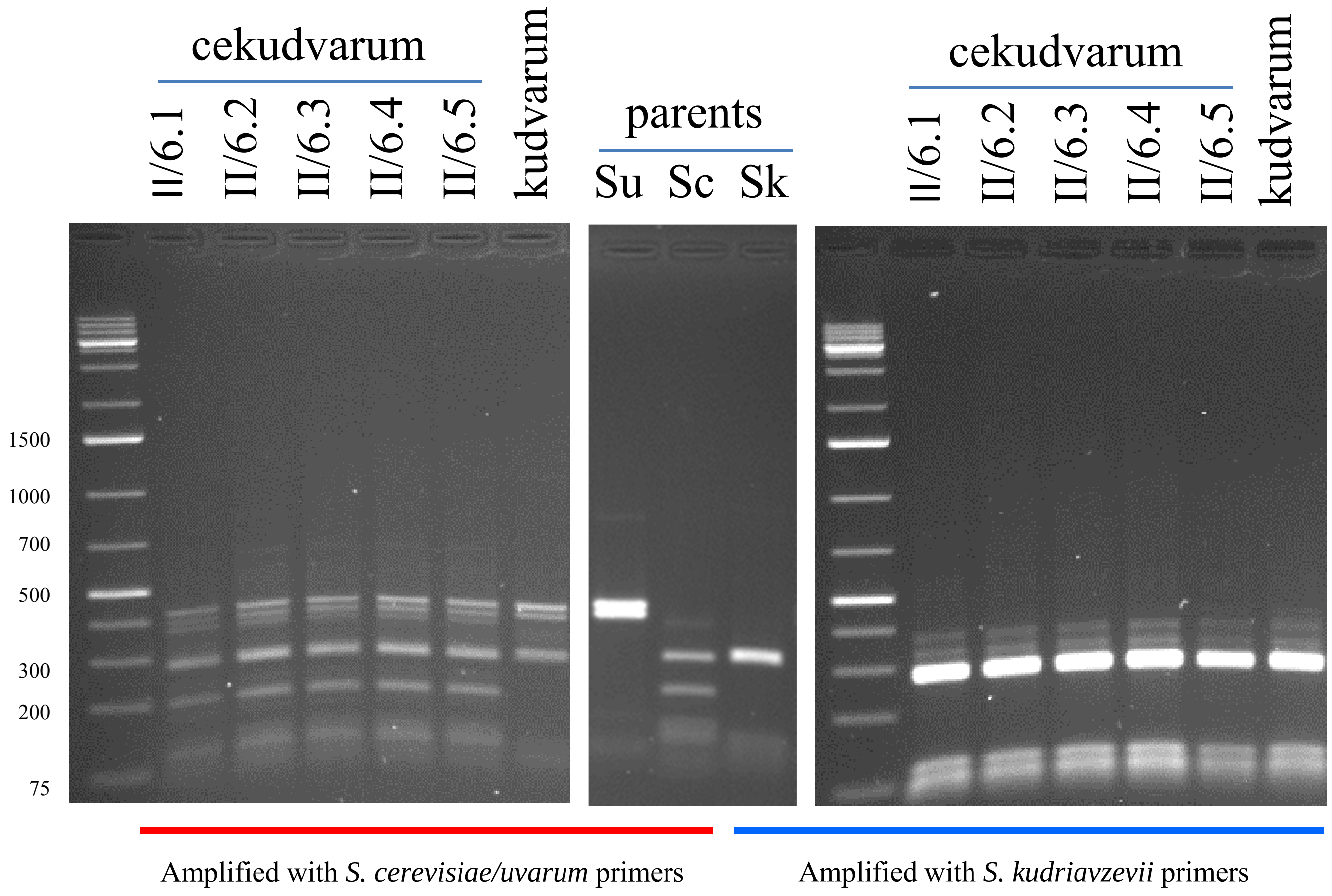

Chromosome XII: *LEU3* digested with *Mse*I. The *S. cerevisiae/uvarum* primers also amplify the *S. kudriavzevii* gene.

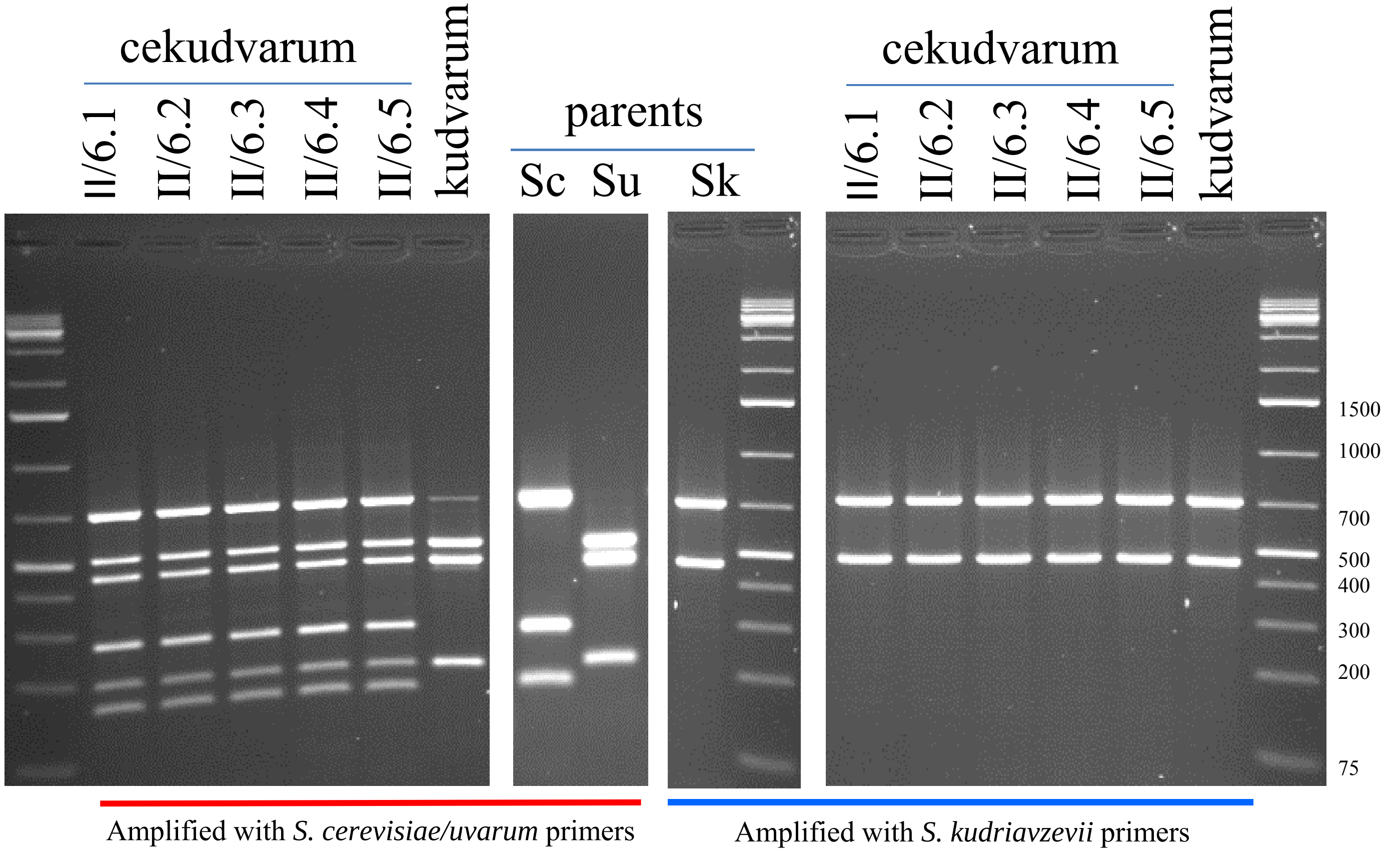

Chromosome XIII: *TDA1* digested with *Hae*III

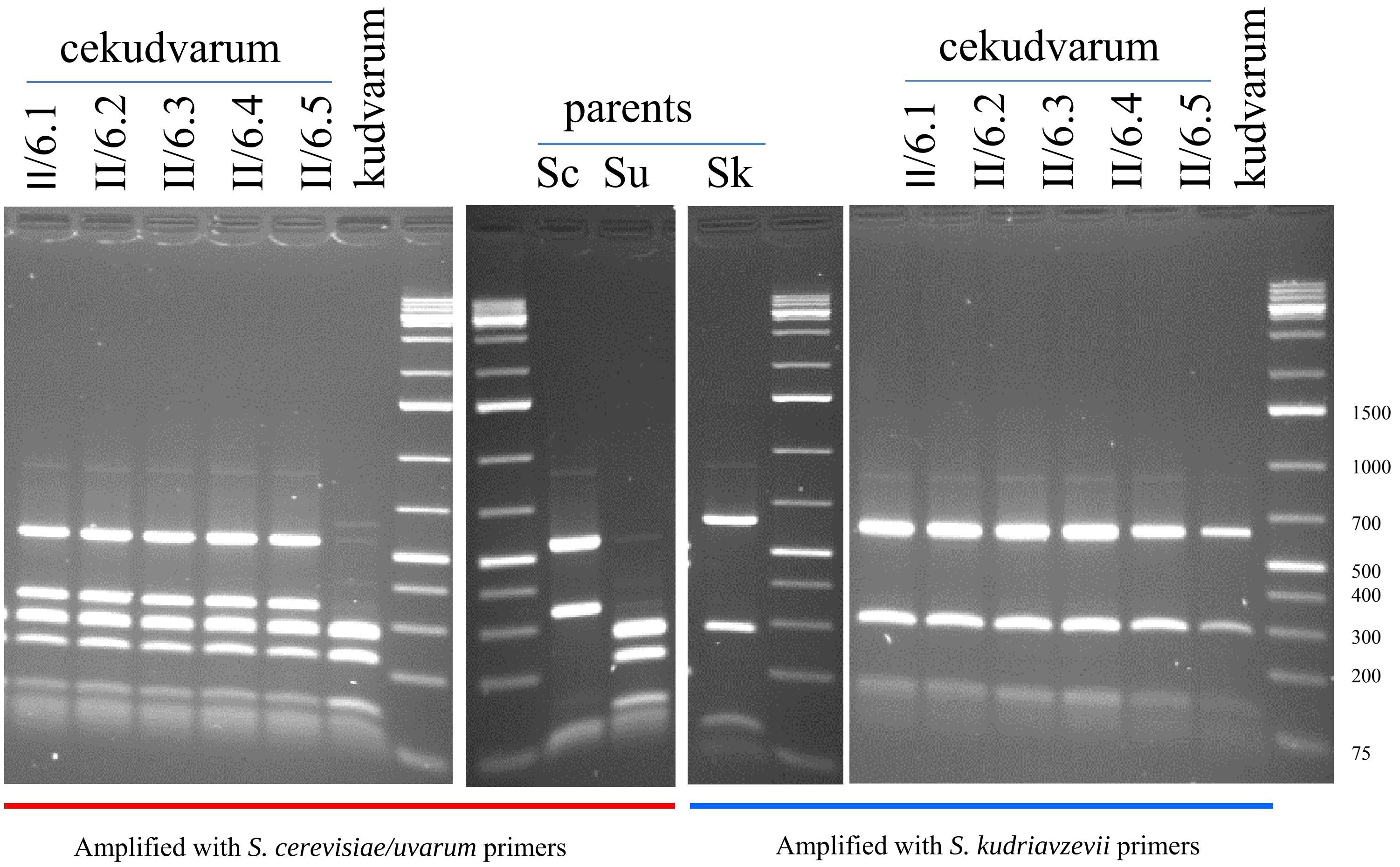

Chromosome XIV: *LRO1* digested with *Rsa*I

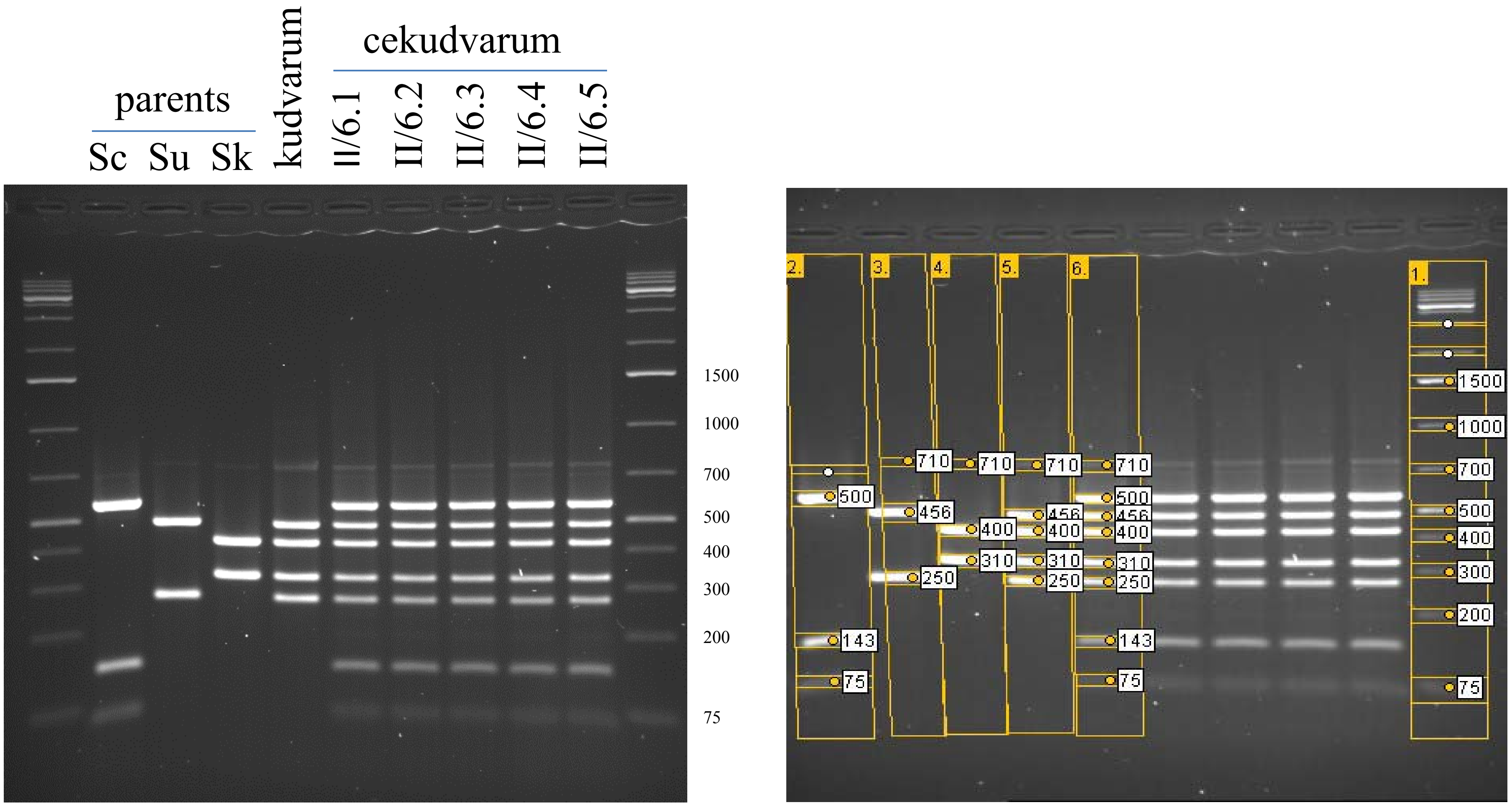

Chromosome XV: *RDR1* digested with *Hae*III. The results of fragment-size determination by gel analysis are also shown.

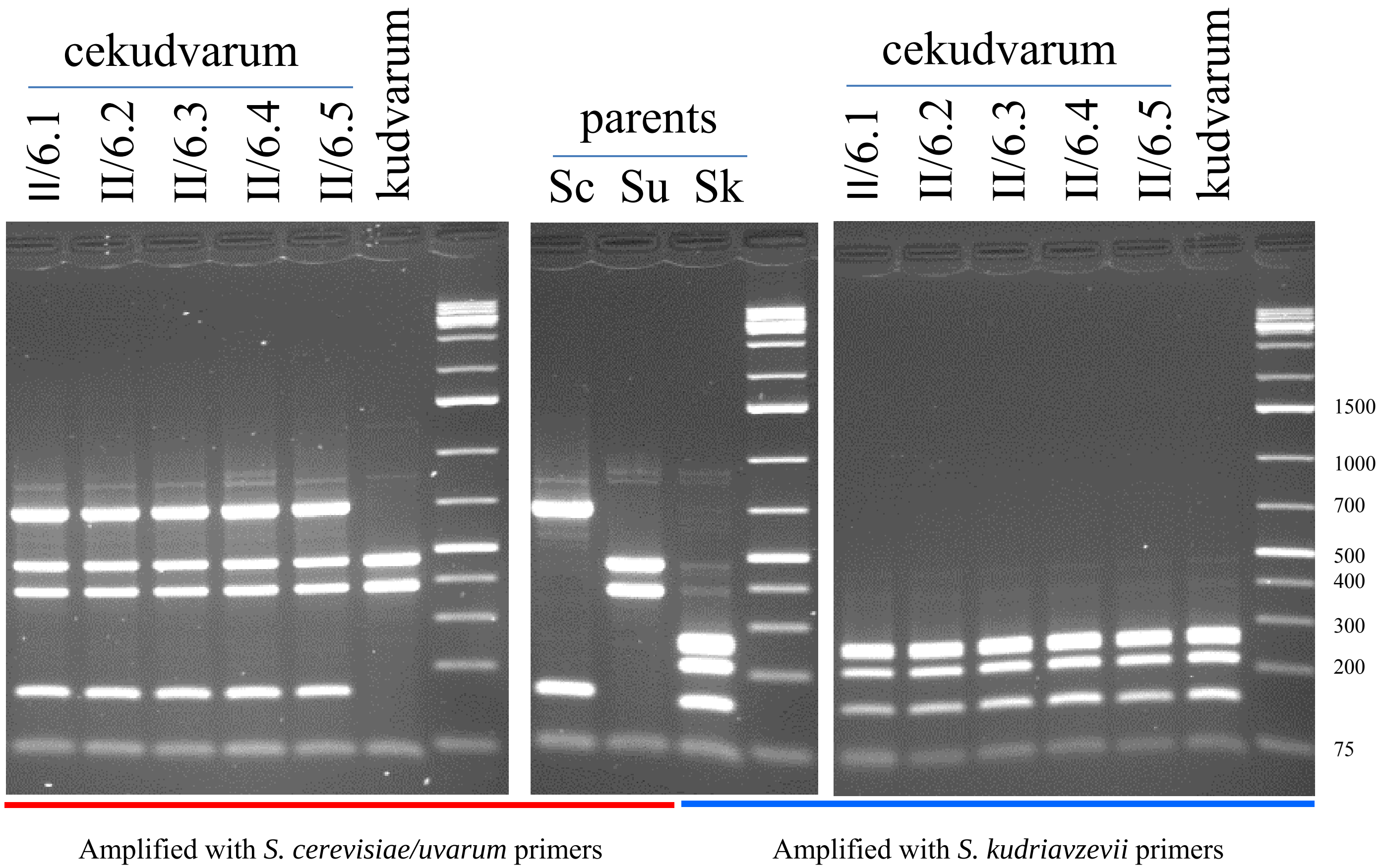

Chromosome XVI: *PRP4* digested with *Hae*III
