# Supplementary material for: Synthetic two-species allodiploid and three-species allotetraploid Saccharomyces hybrids with euploid (complete) parental subgenomes
Source: Sci Rep. 2023 Jan 20;13:1112. doi: 10.1038/s41598-023-27693-2 (PMC9860037; doi:10.1038/s41598-023-27693-2)

Synthetic two-species allodiploid and three-species allotetraploid *Saccharomyces* hybrids with euploid (complete) parental subgenomes

Zsuzsa Antunovics, Adrienn Szabo, Lina Heistinger, Diethard Mattanovich & Matthias Sipiczki

Figure 3S. Sensitivity of strains to 35 °C. Sc, Sk, and Su: *S. cerevisiae*, *S. kudriavzevii* and *S. uvarum* parental strains.

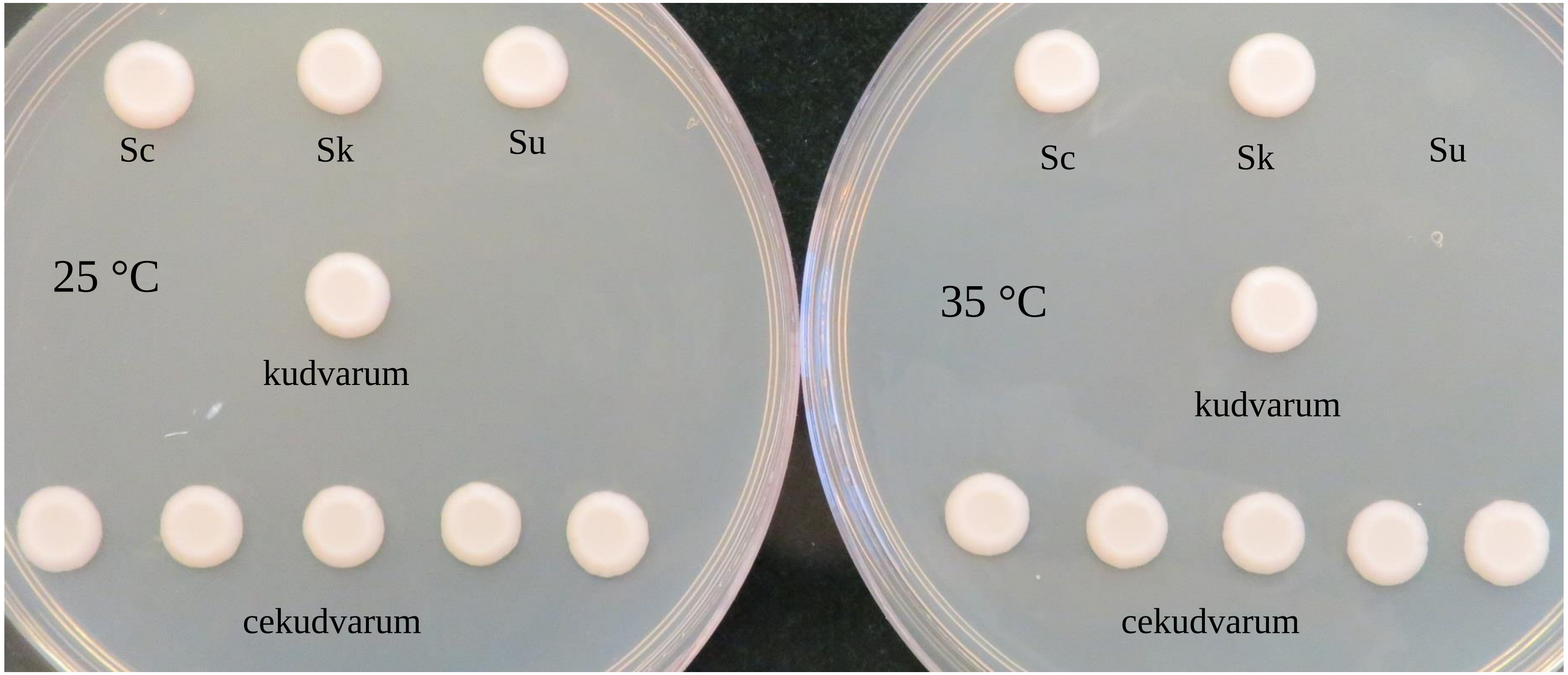

Supplement: Supplementary file 3 — Supplementary Figure S3. [file 41598_2023_27693_MOESM3_ESM.pdf]
