# Supplementary material for: Synthetic two-species allodiploid and three-species allotetraploid Saccharomyces hybrids with euploid (complete) parental subgenomes
Source: Sci Rep. 2023 Jan 20;13:1112. doi: 10.1038/s41598-023-27693-2 (PMC9860037; doi:10.1038/s41598-023-27693-2)

Supplementary Material: Original gel images

Uncropped Figure 1a

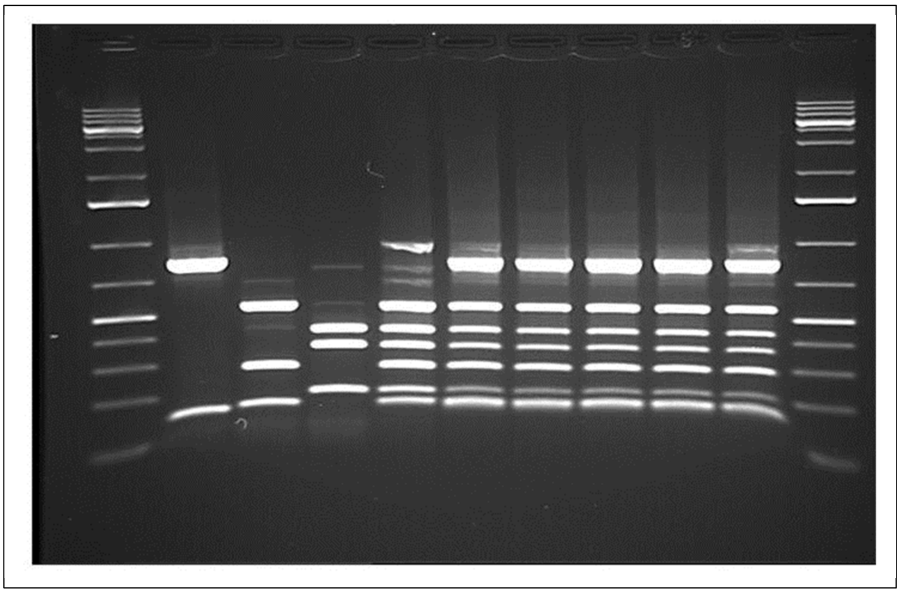

Uncropped Figure 1b

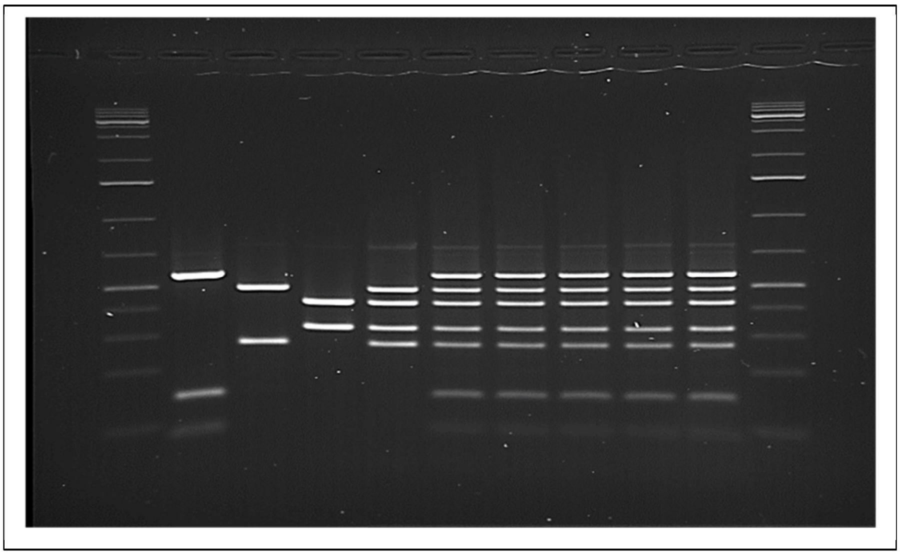

Uncropped Figure 3a

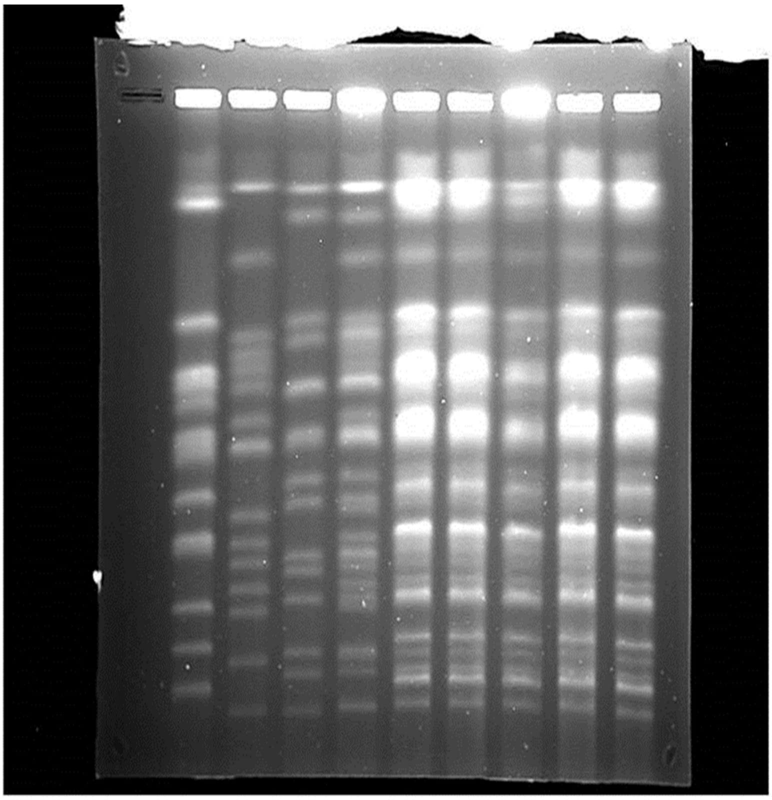

Uncropped Figure 3b

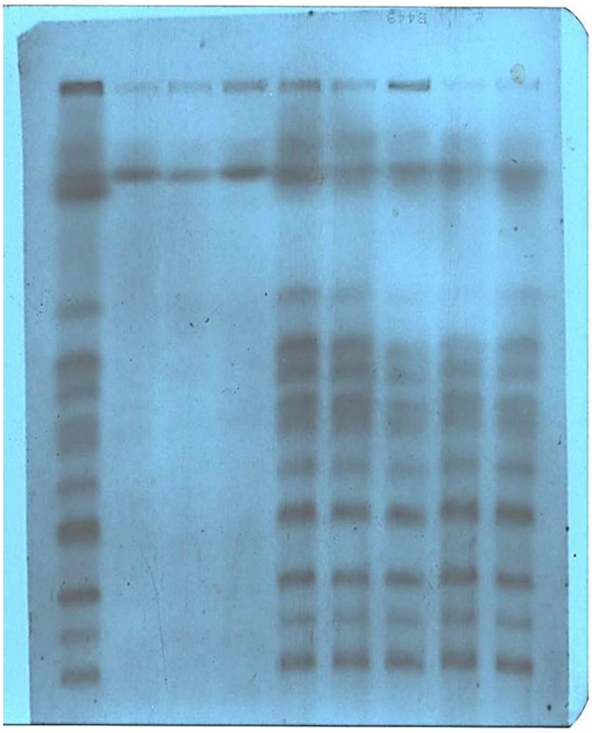

Uncropped Figure 4

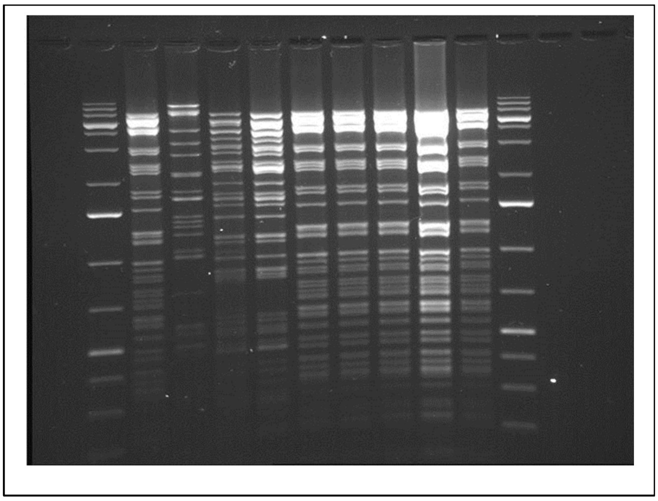

Supplement: Supplementary file 4 — Supplementary Information. [file 41598_2023_27693_MOESM4_ESM.pdf]
