# Supplementary material for: Synthetic two-species allodiploid and three-species allotetraploid Saccharomyces hybrids with euploid (complete) parental subgenomes
Source: Sci Rep. 2023 Jan 20;13:1112. doi: 10.1038/s41598-023-27693-2 (PMC9860037; doi:10.1038/s41598-023-27693-2)
Supplement: Supplementary file 5 — Supplementary Table S1. [file 41598_2023_27693_MOESM5_ESM.pdf]

Synthetic two-species allodiploid and three-species allotetraploid *Saccharomyces* hybrids with euploid (complete) parental subgenomes

Zsuzsa Antunovics, Adrienn Szabo, Lina Heistinger, Diethard Mattanovich & Matthias Sipiczki

Table 1S: Restriction patterns generated by virtual digestion of the amplified fragments with restriction endonucleases

Marker/gene	Restriction endonuclease	Band pattern (bp) *S. cerevisiae*	*S. kudriavzevii*	*S. uvarum*
AFG1	*Rsa*I	372, 364, 80	502, 173, 130	251, 194, 131, 121, 119
ATF1	*Hae*III	635, 235, 40	635, 275	435, 275, 200
BCK2 (Sc/Su)	*Rsa*I	274, 176, 131, 128, 127, 109, 6		590, 361
BCK2 (Sk)			425, 331, 163, 70	
BUD14	*Msp*I	475, 310, 230, 95, 40	720, 310, 110	875, 160, 130
CAT8	*Msp*I	690, 50	450, 205, 80	280, 250, 200
CDC27	*Taq*I	498, 312, 83	677, 128, 85, 72	657, 108, 105, 92
CHS2	*Taq*I	452, 428, 223	710, 223, 170	325, 258, 223, 127, 105, 65
CYR1	*Hind*III	385, 155	540	385, 155
DAL4	*EcoR*V	390, 265, 260, 80	390, 280, 260, 170, 80	890, 390, 330, 280, 180
ECM25	*Rsa*I	337, 319	657, 172	477, 172, 7
FUS1	*Taq*I	342, 269, 262	473, 276, 43	371, 336
GND1(Sc/Su)	*Hinf*I	640, 266		435, 210, 205
GND1(Sk)	*Hinf*I		359, 210, 205. 136, 56	
GND2	*Hae*III	581, 247, 50	528, 272, 50	581, 290,
GSY1	*Taq*I	292, 237, 221, 17	392, 358, 17	441, 309, 17
HCM1	*MspI*	890, 380, 85	500, 430, 400	735, 680
KIN82	*Hinf*I	495, 400, 40	400, 300, 135	700, 130, 95
LEM3	*Hae*III	563, 229, 186, 96, 77	563, 229, 96, 77, 48	818, 74, 65, 21, 10
LEU3 (Sc/Su)	*Mse*I	276, 193, 115, 96, 95, 50, 45, 14, 1		422, 391, 64, 7
LEU3 (Sk)			289, 276, 95, 66, 63, 52, 45, 1	
LRO1 (Sc/Su)	*Rsa*I	553, 336, 82, 58, 11		282, 271, 216, 80, 62, 9
LRO1 (Sk)			618, 282, 99, 60, 13	
MAG2	*Msp*I	480, 400, 110	600, 225, 175	835, 175
MET2	*SacI*	410, 170	410, 170	580
MNT2	*Msp*I	838, 135	429, 356, 188	543, 279, 148
OCA5 (Sc/Su)	*Mse*I	743	405, 331	627, 123
OCA5 (Sk)			462, 405	759, 123
OPY1	*HaeIII*	750	505, 245	405, 345
PRP4 (Sc/Su)	*Hae*III	702, 153, 54		469, 386, 54
PRP4 (Sk)			262, 247, 208, 139, 54	
PTR2	*Hae*III	487, 481, 309, 39	448, 270, 232, 216, 126, 116, 33	498, 448, 171, 120, 36, 33
RDR1	*Hae*III	569, 133, 56, 16	441, 321	489, 266, 16
SAM3	*Hae*III	452, 258, 143, 125, 55, 43	703, 199, 180, 43	1100, 700, 400
SNF1	*Mse*I	291, 237, 219, 47, 7	582, 62	308, 226, 201

STE2	*Hae*III	687, 262	661, 168, 148	511, 262
STE6 (Sc)	*Hae*III	1007, 57		
STE6 (Sk)	*Hae*III		438, 314, 262, 24	
STE6 (Su)	*Hae*III			582, 399
SWH1	*Mse*I	578, 348, 30	484, 233, 55	533, 228, 123, 69
TDA1 (Sc/Sk)	*Hae*III	708, 271, 153, 27		516, 451, 192
TDA1 (Su)			708, 462, 27	
UBP7	*Hinf*I	810, 150	700, 150, 110	405, 405, 160, 76
UGA3 (Sc/Su)	*Rsa*I	438, 322		601, 162
UGA3 (Sk)		458, 251, 103	788, 24	354, 257, 204
